# Transcriptional Basis of Mouse and Human Dendritic Cell Heterogeneity

**DOI:** 10.1016/j.cell.2019.09.035

**Published:** 2019-10-31

**Authors:** Chrysothemis C. Brown, Herman Gudjonson, Yuri Pritykin, Deeksha Deep, Vincent-Philippe Lavallée, Alejandra Mendoza, Rachel Fromme, Linas Mazutis, Charlotte Ariyan, Christina Leslie, Dana Pe’er, Alexander Y. Rudensky

**Affiliations:** 1 Howard Hughes Medical Institute, Memorial Sloan Kettering Cancer Center, New York, NY, USA; 2 Immunology Program, Sloan Kettering Institute, Memorial Sloan Kettering Cancer Center, New York, NY 10065, USA; 3 Infection, Inflammation and Rheumatology Section, UCL Great Ormond Street Institute of Child Health, London WC1N 1EH, UK; 4 Computational and Systems Biology Program, Sloan Kettering Institute, Memorial Sloan Kettering Cancer Center, New York, NY 10065, USA; 5 Ludwig Center at Memorial Sloan Kettering Cancer Center, Memorial Sloan Kettering Cancer Center, New York, NY 10065, USA

**Keywords:** dendritic cells, myeloid cells, T-bet, single-cell RNA-sequencing, ATAC-sequencing, transcriptional regulation

## Abstract

Dendritic cells (DCs) play a critical role in orchestrating adaptive immune responses due to their unique ability to initiate T cell responses and direct their differentiation into effector lineages. Classical DCs have been divided into two subsets, cDC1 and cDC2, based on phenotypic markers and their distinct abilities to prime CD8 and CD4 T cells. While the transcriptional regulation of the cDC1 subset has been well characterized, cDC2 development and function remain poorly understood. By combining transcriptional and chromatin analyses with genetic reporter expression, we identified two principal cDC2 lineages defined by distinct developmental pathways and transcriptional regulators, including T-bet and RORγt, two key transcription factors known to define innate and adaptive lymphocyte subsets. These novel cDC2 lineages were characterized by distinct metabolic and functional programs. Extending our findings to humans revealed conserved DC heterogeneity and the presence of the newly defined cDC2 subsets in human cancer.

## Highlights

• Single-cell analyses reveal novel dendritic cell subsets
• Major cDC2 subsets differentially express T-bet and RORγt
• Distinct pro- and anti-inflammatory potential of T-bet^+^ and Tbet^–^ cDC2s
• Transcriptional basis for cDC2 heterogeneity conserved across mouse and human

## Graphical Abstract

**Figure:**
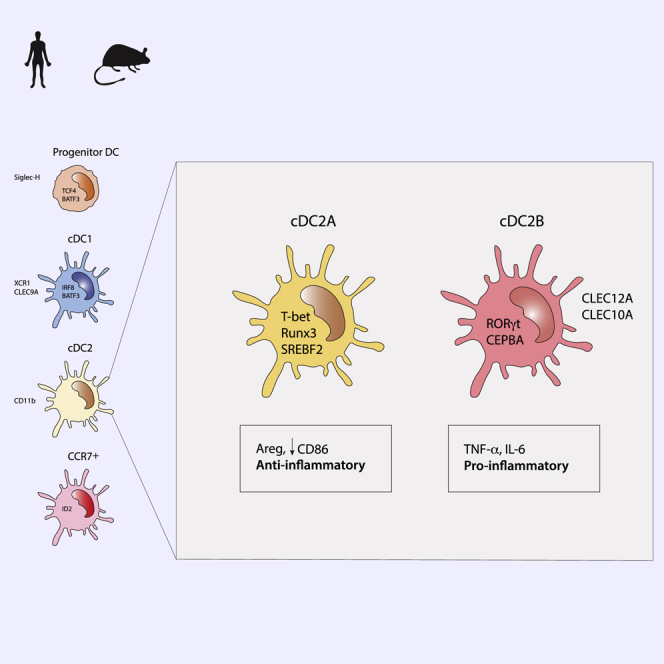

## Teaser

Single-cell analyses of dendritic cells reveals new subsets with distinct pro- and anti-inflammatory potential.

## Introduction

The vast array of infectious and non-infectious challenges faced by the vertebrates ranging from bacteria and viruses to parasites, toxins, and noxious substances requires distinct immune defense strategies reflected in diversification among effector cells of the adaptive immune system, foremost, CD4 T cells. Distinct effector cell subsets, whose functions are defined by divergent repertoires of cytokines and effector molecules tailored to counter corresponding types of threat, emerge upon activation and differentiation of naive CD4 T cells orchestrated by a set of transcription factors (TFs). The latter include T-bet (Tbx21), GATA3, and RORγt promoting differentiation of interferon (IFN)-γ-producing Th1 cells, interleukin (IL)-4-producing Th2 cells, and IL-17-producing Th17 cells, respectively (Kanno et al., 2012, Littman and Rudensky, 2010). Recent discovery of innate lymphocyte ILC1, ILC2, and ILC3 subsets expressing corresponding TF extended the above concept to the innate lymphoid cell counterparts of CD4 T cells (Colonna, 2018).

Dendritic cells (DC) sense microbial agents and direct elaboration of protective T cell responses commensurate to the challenge type. Classical DCs (cDCs), defined by the expression of integrin-αX (CD11c) and major histocompatibility complex class II (MHC class II) (Steinman et al., 1979), comprise two subsets, cDC1s and cDC2s (Guilliams et al., 2014). This initial division reflects developmental and functional heterogeneity among DCs. cDC1s, identified by cell surface expression of XCR1, CD8α, CLEC9A, or CD103 (Durai and Murphy, 2016), are developmentally dependent on IRF8 and BATF3 (Aliberti et al., 2003, Hildner et al., 2008). cDC1s cross-present antigens and prime cytotoxic CD8^+^ T cell responses to intracellular pathogens (Dudziak et al., 2007, Hildner et al., 2008, Yamazaki et al., 2013). Initial studies characterizing human DCs using single-cell transcriptomics indicate that cDC1s are a relatively homogeneous population (Villani et al., 2017). By contrast, cDC2s, defined by the cell surface expression of CD11b and CD172α (Durai and Murphy, 2016), comprise a heterogeneous population of cells with differential surface expression of Esam, Mgl2 (CD301b), or CLEC12A (Kumamoto et al., 2013, Lewis et al., 2011). These DC sub-types exhibit variable dependence on IRF4 and Notch signaling (Lewis et al., 2011, Schlitzer et al., 2013) and appear to have distinct functional roles (Gao et al., 2013, Kashem et al., 2015, Kumamoto et al., 2016, Linehan et al., 2015), suggesting further diversification within cDC2s. However, our understanding of cDC2 heterogeneity and its biological implications has been limited by a lack of knowledge of its transcriptional basis, required for the development of genetic tools to selectively target DC subsets.

Here, we sought to explore DC heterogeneity and its transcriptional basis. Using single-cell, “bulk” RNA sequencing (RNA-seq), assay for transposase-accessible chromatin using sequencing (ATAC-seq), and specific gene reporter analyses, we systematically characterized all known DC subsets. In an unexpected parallel to innate and adaptive lymphoid cell subsets, we uncovered two overarching cDC2 subsets defined by expression of a distinct set of TFs, including T-bet and RORγt. Acquisition of opposing cDC2 fates accompanied by induction of these TFs in response to environmental cues forms the basis of their phenotypic and functional heterogeneity, conserved across mice and humans.

## Results

### Unbiased Dissection of DC Subsets by Single-Cell RNA-Seq

**Figure 1:**
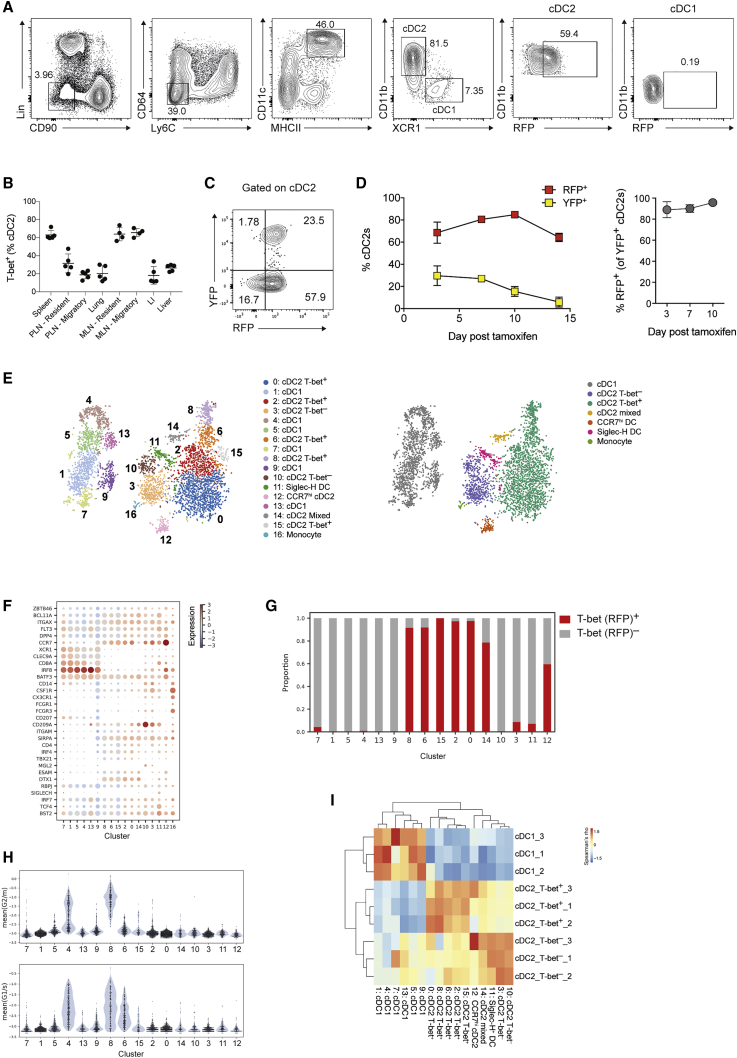
Single-Cell Survey Reveals Heterogeneity of cDC2s with Two Subsets Delineated by Expression of T-Bet (A) Representative contour plot showing gating strategy for splenic DCs in *Tbx21*^*RFP-Cre*^ mice. DCs defined as Lin(CD3,CD19,CD49b,Siglec-F)^–^Ly6C^–^CD64^–^CD11c^+^MHCII^+^. (B) Frequency of T-bet^+^ cDC2s across tissues. Each circle represents one mouse. In the peripheral and mesenteric LN (PLN and MLN), migratory DCs were defined as MHCII^hi^CD11c^int^ and resident DCs as MHCII^int^CD11c^hi^. Error bars represent mean ± SEM. (C) Analysis of RFP^+^ and YFP^+^ splenic cDC2s from *Tbx21*^*RFP-CreERT2*^*Rosa26*^*YFP*^ mice, 3 days post tamoxifen gavage. (D) Percent RFP^+^ and YFP^+^ of cDC2 cells. Percent RFP^+^ of YFP^+^ cDC2s at indicated time points post tamoxifen gavage (right). Error bars represent mean ± SEM; n = 3–4 mice per time point. (E) t-SNE embedding of 4,464 DCs. Colors indicate unsupervised clustering by Phenograph (left panel) or classification based on expression of canonical markers (right panel). (F) Expression of canonical DC markers across the transcriptionally defined DC clusters from (E). (G) Proportion of T-bet (RFP^+^) cells in each cell cluster identified in (D). (H) Violin plot showing expression of the cell-cycle signature across the DC clusters from (E). (I) Similarity of bulk T-bet^–^ cDC2s, T-bet^+^ cDC2, and cDC1 transcriptomes to the reference single-cell DC clusters (E). Colors represent the correlation coefficient between the cell population identified in the row label and the DC cluster identified by the column label. See also Figures S1 and S7.

**Figure S1:**
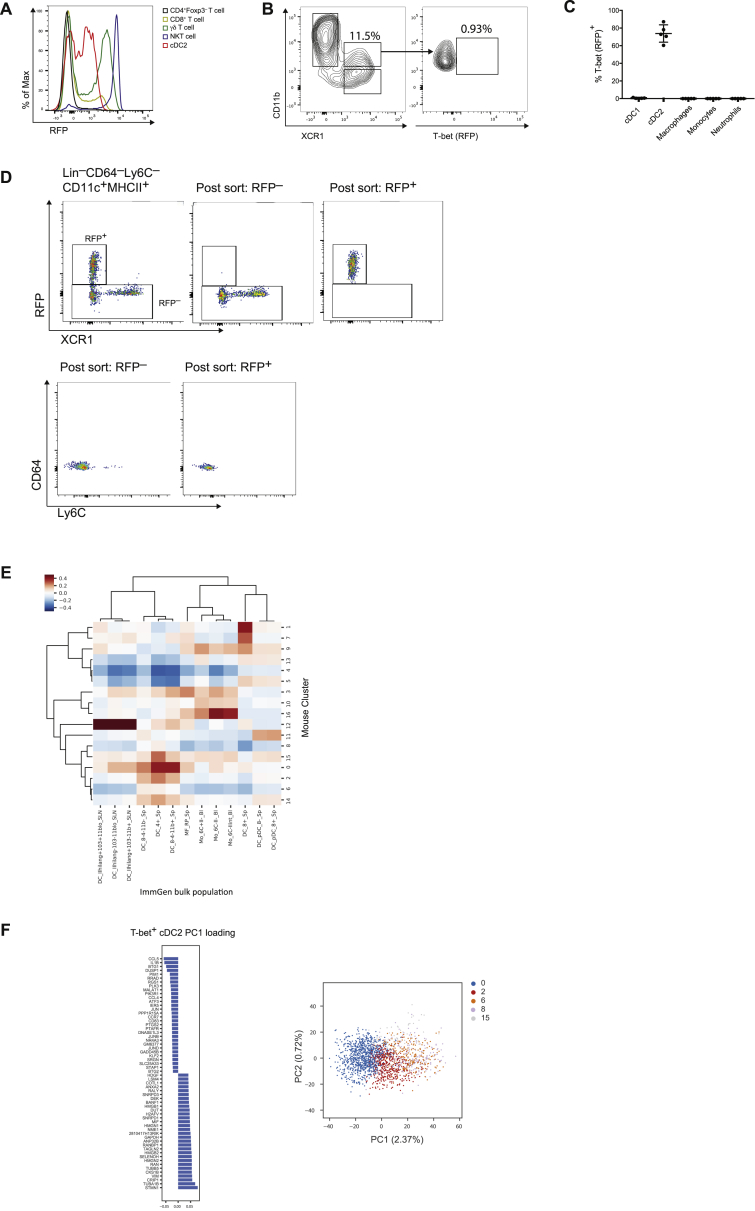
Single-Cell Survey Reveals Heterogeneity of cDC2s, Related to Figure 1 A. Representative histogram showing expression of T-bet (RFP) in splenic cells from *Tbx21*^*RFP-cre*^ mice. (B). Expression of T-bet in CD11b^+^XCR1^+^ DCs from the intestinal lamina propria. Data representative of > 5 independent experiments, with at least 3 mice per experiment. (C). Expression of T-bet in splenic myeloid cells. Cells were defined as: (i) Ly-6C^hi^ monocytes (Lin ^–^Ly6C^+^Ly6G^–^CD11b^+^CX3CR1^+^); neutrophils (Lin^–^Ly6C^+^Ly6G^+^); macrophages (Lin^–^CD64^+^Ly6C^–^). Lineages (Lin) were defined as: CD3e, CD90.2, CD19, CD49b and Siglec F. Each circle represents an individual mouse, error bars represent mean ± SEM. (D). Left: Gating strategy for single-cell sorting. DCs were defined as Lin(CD3, CD19, CD90)^–^Ly6C^–^CD64^–^CD11c^+^MHCII^+^. Two populations were sampled: RFP^+^ DCs and RFP^–^ DCs (encompassing XCR1^+^ cDC1s, CD11b^+^RFP^–^ and CD11b^–^XCR1^–^ DCs). Right: Post-sort purity of RFP^+^ and RFP^–^ cells. Contaminating population of Ly6C^+^ cells identifiable on post-sort purity (lower panel). (E). Similarity of splenic CD11c^+^MHCII^+^ cells to reference myeloid cells (ImmGen Consortium) Colors represent the Pearson correlation between the mean gene expression from the dendritic cell cluster in the rows and the bulk reference transcriptome in the columns. (F). Top 20 positive and negative gene loadings of PC1 for T-bet^+^ cDC2 clusters after cell-cycle correction (left panel). Scatterplot of PC1 and PC2 for T-bet^+^ cDC2 clusters after cell-cycle correction (right panel).

The transcription factor T-bet delineates subsets of innate lymphoid cells (ILCs) and T helper cells with distinct effector programs, and its expression has also been reported in DCs (Lugo-Villarino et al., 2003). To comprehensively analyze T-bet expression across splenic DCs, we utilized a T-bet reporter allele, *Tbx21*^*RFP*^^*-Cre*^, which faithfully reports endogenous T-bet protein expression (Levine et al., 2017). This revealed that T-bet was uniquely expressed in a subset of CD11b^+^XCR1^–^ cDC2s in all lymphoid and mucosal tissues examined (Figures 1A, 1B, and S1A). Of note, we did not identify T-bet^+^ cDC2s within the intestinal CD11b^+^XCR1^+^ (DP) cDC subset (Figure S1B). T-bet was not expressed in other myeloid cell lineages (Figure S1C). Genetic fate mapping by administration of tamoxifen to *Tbx21*^*RFP-CreERT2*^*Rosa26*^*YFP*^ mice revealed that DCs that expressed T-bet at the time of Cre-mediated YFP tagging, retained its expression over their lifespan (Figures 1C and 1D). Thus, T-bet-expressing cDC2s represent a stable cell lineage. History of T-bet expression marked by YFP was not detectable in cDC1s (data not shown) indicating that T-bet expression is acquired after DC progenitors commit to cDC2 cell fate. These results suggested that cDC2s may harbor additional subsets defined by expression of alternative TFs.

To uncover the full spectrum of DC heterogeneity, we utilized droplet-based single-cell RNA-sequencing (scRNA-seq) to profile splenic DCs defined as Lin(CD3,CD19,CD90)^–^Ly6C^–^CD64^–^MHCII^+^CD11c^+^. Given previous reports of poor *Tbx21* transcript detection using such methods (Bernink et al., 2017, Björklund et al., 2016), we elected to profile fluorescence-activated cell sorting (FACS) purified RFP(T-bet)^+^ and RFP(T-bet)^–^ DCs separately (Figure S1D) to enable definitive post hoc identification of T-bet-expressing cells. A total of 4,464 single-cell transcriptomes were generated after pre-processing. Analysis of gene expression was performed on the compiled data from the two populations without consideration of cell-surface markers utilized for sorting. We performed unsupervised graph clustering using Phenograph (Levine et al., 2015), opting for a finer clustering of the data to increase the sensitivity of our analysis to small sub-populations. This identified 17 distinct clusters, visualized using t-distributed stochastic neighbor embedding (t-SNE) (Figure 1E). Comparison of their transcriptional features with immune cell transcriptome profiles reported by ImmGen Consortium (Miller et al., 2012) revealed a contaminating population of Ly6C^+^ monocytes (cluster 16; Figures S1D and S1E) that were removed from downstream analyses. We established the cell identity of each cluster through the analysis of canonical DC gene expression (Figure 1F), similarity with bulk transcriptomes from Immgen datasets (Figure S1E) and proportion of RFP(T-bet)^+^ cells for each cluster (Figure 1G). CLEC9A^+^XCR1^+^ cDC1s were partitioned across 6 clusters (1, 4, 5, 7, 9, and 13), T-bet^+^CD11b^+^SIRPα^+^ cDC2s were represented by 5 clusters (0, 2, 6, 8, 15) and T-bet^–^CD11b^+^SIRPα^+^ cDC2s by 2 clusters (3 and 10). In agreement with a recent study (Cabeza-Cabrerizo et al., 2019), we identified proliferating DCs marked by enrichment of genes associated with cell-cycle comprising 19.1% of cDC1s (cluster 4) and 15.7% of cDC2s (clusters 6 and 8; Figure 1H), suggesting that mature splenic DCs are actively dividing in the steady state. After removal of cell-cycle signals, the first principal component of variation which drives the phenotypic diversity in T-bet^+^ cDC2s comprises genes associated with DC maturation (*Cd83*, *Ccr7*), along with increased expression of cytokines and chemokines (Figure S1F). Thus, the clusters of T-bet^+^ cDC2s likely represent a continuum of discrete states of maturation rather than separate phenotypic entities. Remaining heterogeneity among cDC1 clusters was primarily accounted for by cells in cluster 9, which exhibited a mixed cDC1/cDC2 phenotype and characteristics of “doublets,” potentially arising from phagocytosis. Cluster 14 contained a mixture of T-bet^+^ and T-bet^–^ cDC2s. Two clusters (11 and 12), lacking both *Itgam* (CD11b) and *Xcr1*, did not align with either cDC1s or cDC2s. Cluster 11 cells had a weak cDC2 signature (Figure 1F) while also expressing genes associated with plasmacytoid DCs (*Bst1*, *Il3ra*, *Siglech*, and *Irf7*) (Figure 1F), reminiscent of recently described circulating human pre-DCs (See et al., 2017, Villani et al., 2017). Cluster 12 cells were distinguished by high *Ccr7* expression and a gene signature that correlated with peripheral lymph node (LN) MHCII^hi^ “migratory” DCs (Figure S1E; Miller et al., 2012). The transcriptional profiles of the clusters identified by scRNA-seq analyses closely resembled those of bulk-sorted T-bet^–^ cDC2s, T-bet^+^ cDC2s, and cDC1s analyzed using conventional RNA-seq (Figure 1I).

### Characterizing cDC2 Heterogeneity

**Figure 2:**
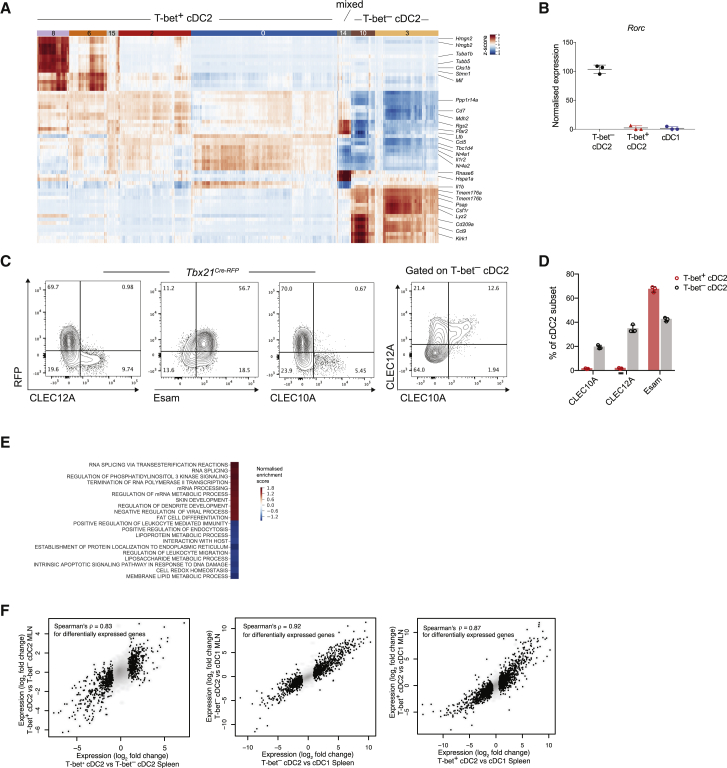
T-bet and RORγt Expression Delineates Distinct cDC2 Subsets (A) Heatmap reporting scaled, imputed expression for the top 10 differentially expressed genes for each cDC2 cluster identified in Figure 1E. Genes of interest are shown on the right. (B) Expression of *Rorc* transcript in bulk sorted T-bet^–^ cDC2s, T-bet^+^ cDC2s, and cDC1s. (C) Expression of CLEC12A, Esam, and CLEC10A in cDC2s from *Tbx21*^*RFP-Cre*^ mice. Far right: CLEC12A and CLEC10A expression within T-bet^–^ cDC2s. (D) Summary graph for (C). Each symbol represents one mouse. (E) Enrichment of GO pathways in in T-bet^+^ versus T-bet^–^ cDC2 clusters. (F) Correlation between splenic and MLN cDC2 transcriptomes. Error bars represent mean ± SEM. See also Figures S2 and S7 and Tables S1, S2, and S3.

**Figure S2:**
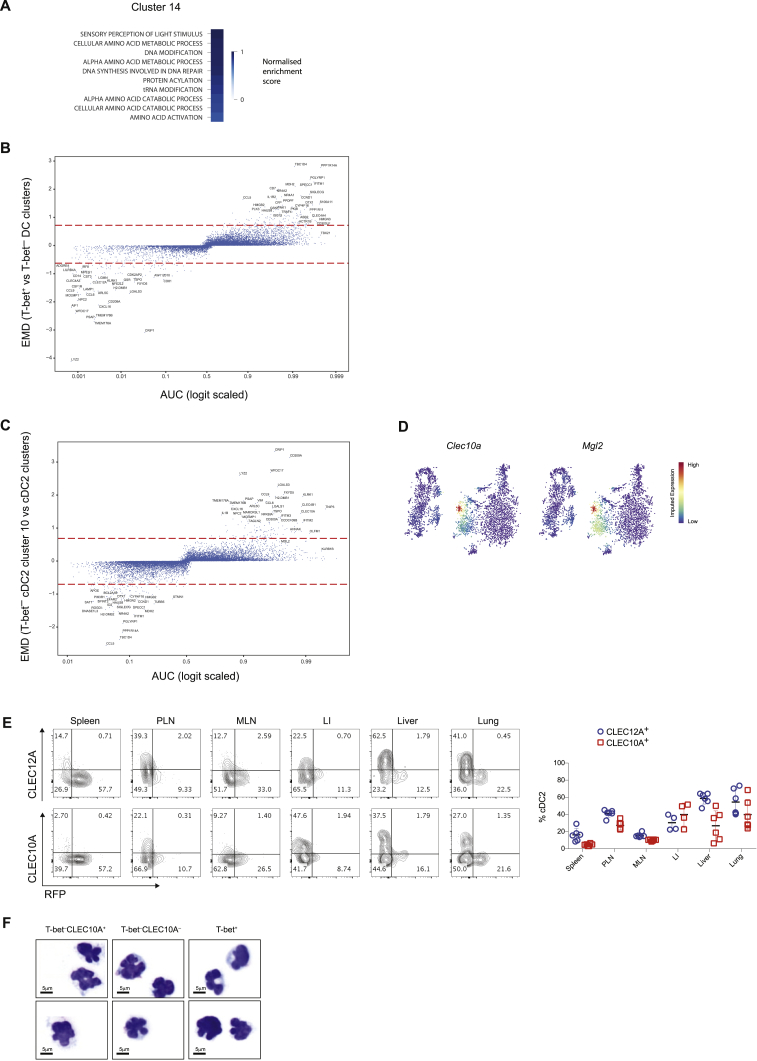
Distinct cDC2 Subsets Express Divergent Transcription Factors and Cell Surface Markers. Related to Figure 2 (A). Enrichment of GO pathways (biological processes) in cluster 14. (B). Graph showing AUC score (x axis) for genes differentially expressed between T-bet^+^ and T-bet^–^ cDC2 clusters. Earth movers distance (EMD) on the y axis. Dashed lines represent μ_EMD_ ± 3σ_EMD_. (C). Graph showing AUC score (x axis) for genes differentially expressed between T-bet^–^ cluster 10 and all other cDC2 clusters. EMD on the y axis. Dashed lines represent μ_EMD_ ± 3σ_EMD_. (D). t-SNE map of splenic DCs colored by imputed expression of *Clec10a* and *Mgl2* demonstrating co-expression of these genes by cells in cluster 10. (E). Representative flow cytometry plots showing expression of CLEC12A, CLEC10A and T-bet (RFP) in cDC2s isolated from spleen, peripheral lymph nodes (PLN), mesenteric lymph nodes (MLN), large intestine lamina propria (LI), liver and lung (left). Frequency of CLEC12A^+^ and CLEC10A^+^ cells within all cDC2s (right). Each symbol represents a single mouse. Data representative of 2 independent experiments. (E). Cytospin analysis of Wright-Giemsa stained sorted cDC2 subsets.

To gain further insight into cDC2 heterogeneity, we identified differential gene expression signatures for the individual cDC2 clusters (Figure 2A; Table S1). Shared gene expression among individual T-bet^+^ cDC2 clusters suggests that these clusters represent a single cellular population with graded gene expression states. Cluster 14, comprising T-bet^+^ and T-bet^–^ cDC2s, was distinguished by an increased metabolically active state (Figure S2A). The two T-bet^–^ cDC2 clusters (3 and 10) shared expression of *Csf1r* and *Lyz2* and other genes known to be associated with monocytes and macrophages (Figure 2A). *Tmem176a* and *Tmem176b* were highly expressed in T-bet^–^ cDC2s (Figure 2A). This observation was particularly intriguing given that expression of these genes is RORγt-dependent and a feature of all RORγt lymphocytes—ILC3s and Th17 cells (Ciofani et al., 2012, Drujont et al., 2016, Robinette et al., 2015). Although we failed to detect expression of *Rorc* by scRNA-seq (data not shown), it was highly enriched in T-bet^–^ cDC2s in the bulk RNA-seq dataset (Figure 2B). Collectively, these data identify two broad cDC2 subsets defined by the mutually exclusive expression of T-bet or RORγt.

To identify discriminative cell surface markers that would best classify cells into T-bet^+^ cDC2s or T-bet^–^ cDC2s, we combined the earth mover's distance (EMD)-based measure of differential expression with a measure of classification—the area under the ROC curve (AUC) metric—on imputed expression values (McClish, 1989). Among 64 genes identified (AUC ≥ 0.99, EMD ≥ 4 SD; Figure S2B; Table S2), T-bet^+^ cDC2s were best defined by the expression of the Notch target *Dtx1* indicating that this subset may represent the Notch2-dependent cDC2 subset, previously distinguished by cell surface expression of Esam (Lewis et al., 2011). T-bet^–^ cDC2 clusters uniquely expressed CLEC12A and could be further separated based on the expression of several cell surface markers by cluster 10 cells including CLEC10A (CD301a), CD209a, and NKG2D (*Klrk1*) (Figures 2A and S2C). *Cd301b* (Mgl2) was exclusively expressed by this cluster (Figure S2D) indicating that Mgl2^+^ cDC2s, previously reported in the skin and draining LN (Kumamoto et al., 2013), are akin to T-bet^–^ cDC2.

Flow cytometric analysis confirmed that CLEC12A was exclusively expressed by T-bet^–^ DCs (Figures 2C and 2D). Esam alone was not sufficient to discriminate between T-bet^+^ and T-bet^–^ DCs (Figures 2C and 2D). In agreement with the scRNA-seq analysis, CLEC10A expression was limited to a subset of (T-bet^–^/CLEC12A^+^) cDC2s (Figures 2C and 2D). Further analysis of lymphoid and non-lymphoid tissue confirmed that CLEC12A^+^ and CLEC10A^+^ cDC2 subsets reside within the T-bet^–^ cDC2 fraction (Figure S2E). Thus, a combination of Esam and CLEC12A could be used to distinguish T-bet^+^ DCs from T-bet^–^ DCs with CLEC10A further delineating two subsets of T-bet^–^ DCs. Using these newly defined markers we sorted T-bet^+^, T-bet^–^CLEC10A^+^, and T-bet^–^CLEC10A^–^ cDC2 subsets and assessed their morphology using cytospin. These cell subsets were morphologically indistinguishable with classic cerebriform nuclei typical of DCs (Figure S2F; Inaba et al., 1992, Villani et al., 2017).

Further examination of unique transcripts that discriminated T-bet^–^ DCs from T-bet^+^ counterparts revealed markedly increased expression of *Psap*, encoding a precursor for saposins, and *Npc2*, which regulates the transport of cholesterol from lysosome (Infante et al., 2008; Figures 2A and S2B). Furthermore, gene set enrichment analysis (GSEA) analysis revealed enrichment of pathways involved in lipid localization, transport, and metabolism in T-bet^–^ cDC2s (Figure 2E). In contrast, T-bet^+^ cDC2s were characterized by high levels of *Tbc1d4*, a gene regulating glucose transporter expression (Eguez et al., 2005) and *Mdh*, an enzyme that supports NADH recycling and sustained glycolysis (Figure S2B; Gaude et al., 2018). Thus, metabolic properties of these subsets appear distinct.

Transcriptional profiles of T-bet^+^ and T-bet^–^ cDC2s, as well as cDC1s isolated from spleen or mesenteric LN (MLN) using bulk RNA-seq revealed near perfect correlation of differentially expressed DC subset-specific genes across tissues (Figure 2F). A core set of 69 genes, including *Tbx21*, *Dtx1*, and *Ccr6*, was overexpressed by all T-bet^+^ cDC2s irrespective of their tissue location. Genes that defined T-bet^–^ cDC2s included *Clec12a*, *Cx3cr1*, *Cd14*, *Il1a*, and *P2rx7* (Table S3). A number of the cDC2 subset defining genes have not previously been associated with DC differentiation or function.

### Relationship of cDC2 Subsets to Monocytes

**Figure 3:**
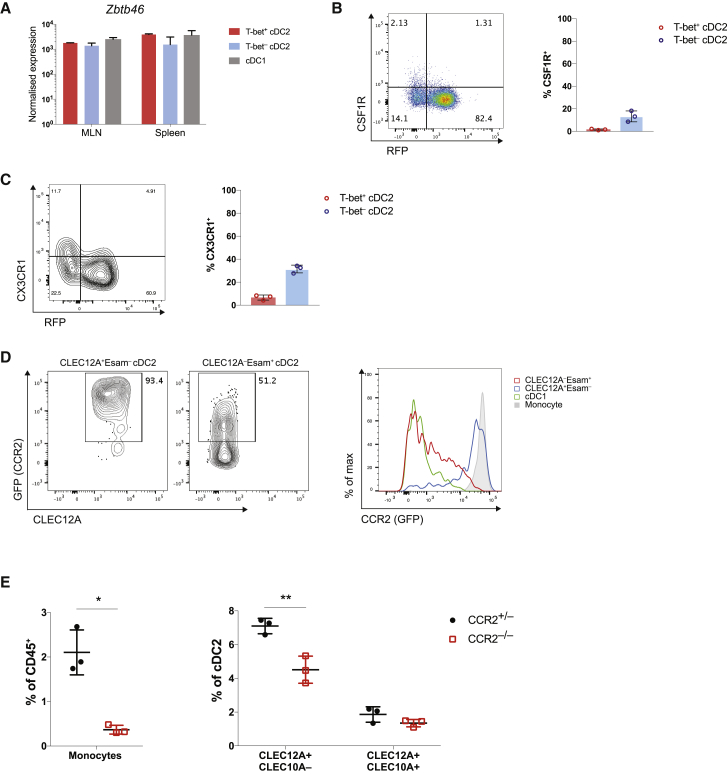
T-bet^–^ cDC2s Are Distinct from Monocytes (A) Expression of *Zbtb46* in bulk sorted DC subsets. (B) Representative plot showing expression of CSF1R in cDC2s and summary graph (right). Each symbol represents one mouse. (C) Representative plot showing expression of CX3CR1 in cDC2s and summary graph (right). Each symbol represents one mouse. (D) Expression of CCR2 (GFP) in splenic DCs. T-bet^–^ cDC2s identified as CLEC12A^+^Esam^–^ and T-bet^+^ cDC2s as CLEC12A^+^Esam^–^ cDC2s. Monocytes gated as Lin^–^Ly6C^+^CD11b^+^CX3CR1^+^. Each symbol represents one mouse. (E) Frequency of monocytes and indicated cDC2 subset in CCR2^–/–^ mice and CCR2^+/–^ littermates. Error bars represent mean ± SEM; p values calculated using Student’s t test.

T-bet^–^ DCs expressed higher levels of genes previously associated with monocytes including *Csf1r*, *Ccr2*, and *Cx3cr1*. Analysis of Zbtb46 expression, known to distinguish cDCs from monocytes and macrophages (Meredith et al., 2012, Miller et al., 2012, Satpathy et al., 2012), demonstrated similar levels of expression between T-bet^+^ and T-bet^–^ cDC2s (Figure 3A), confirming their identity as cDCs. Furthermore, analysis of monocyte markers showed that while T-bet^–^ cDC2s expressed higher levels of Csf1r mRNA, only a small subset of T-bet^–^ cDC2s expressed CSF1R protein (Figure 3B). CX3CR1 was expressed by ∼30% of T-bet^–^ cDC2s (Figure 3C), consistent with a previous study of cDC-enriched CX3CR1^+^ antigen-presenting cells (Esterházy et al., 2016). Analysis of *Ccr2*^*GFP*^ mice revealed higher CCR2 by CLEC12A^+^ Esam^–^ (T-bet^–^) than CLEC12A^–^Esam^+^(T-bet^+^) cDC2s. Nevertheless, ∼50% of T-bet^+^ cDC2s were also CCR2^+^ (Figure 3D). Finally, we assessed the impact of CCR2 ablation on T-bet^–^ cDC2s. Whereas *Ccr2* deletion led to loss of monocytes as expected (Serbina and Pamer, 2006), CLEC10A^+^T-bet^–^ cDC2s were unaffected and CLEC10A^–^T-bet^–^ cDC2s showed only a partial reduction in frequency (Figure 3E). Thus, the currently accepted “monocyte” gene signature is not sufficient to discriminate between monocytes and T-bet^–^ cDC2s due to considerable overlap in gene expression across these cell types. Instead, steady-state monocytes and cDC2s can be distinguished by exclusion of Ly6C^+^ cells in agreement with a recent scRNA-seq analysis of MHC class II^+^ monocytes (Mildner et al., 2017). Shared expression of genes such as CX3CR1, CCR2, and CSF1R may thus be a reflection of close relatedness of cDCs and monocytes with shared phenotypes and functions.

### Transcriptional Regulation of Chromatin Accessibility and Gene Expression in cDC2s

**Figure 4:**
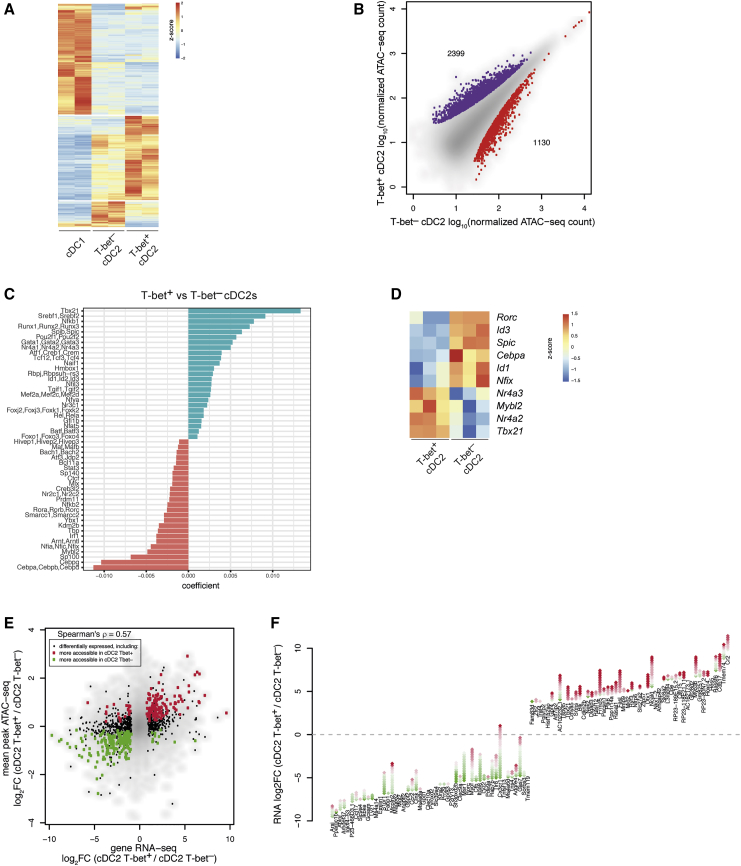
Transcriptional and Epigenetic Landscape of cDCs (A) Heatmap showing differentially accessible ATAC-seq peaks in cDC1s, T-bet^+^ cDC2s, and T-bet^–^ cDC2s. Color bar, accessibility *Z* score. (B) ATAC-seq analysis of chromatin accessible sites in cDC2s. Peaks shown in purple showed increased read count in T-bet^+^ cDC2s, peaks shown in red were enriched in T-bet^–^ cDC2s. (C) Predictive value of TF motifs in peaks more accessible in T-bet^–^ cDC2s (red) or T-bet^+^ cDC2s (green). (D) Heatmap reporting scaled expression of predicted transcriptional regulators in T-bet^–^ cDCs versus T-bet^+^ cDC2s. Color bar, accessibility *Z* score. (E) Correlation between average differential accessibility of peaks associated with a gene and gene expression in T-bet^+^ versus T-bet^–^ cDC2s. (F) Diamond plot showing gains and losses of regulatory elements for top 20 most differentially expressed genes in T-bet^+^ versus T-bet^–^ cDC2s. Each diamond represents a chromatin accessibility peak associated with the indicated gene. Green denotes ATAC-seq peaks that gained accessibility in T-bet^+^ cDC2s, red diamonds denote peaks that lost accessibility. The bottom-most peak on the y axis corresponds to the log_2_FC in differential expression of the gene. See also Figure S3 and Table S4.

**Figure S3:**
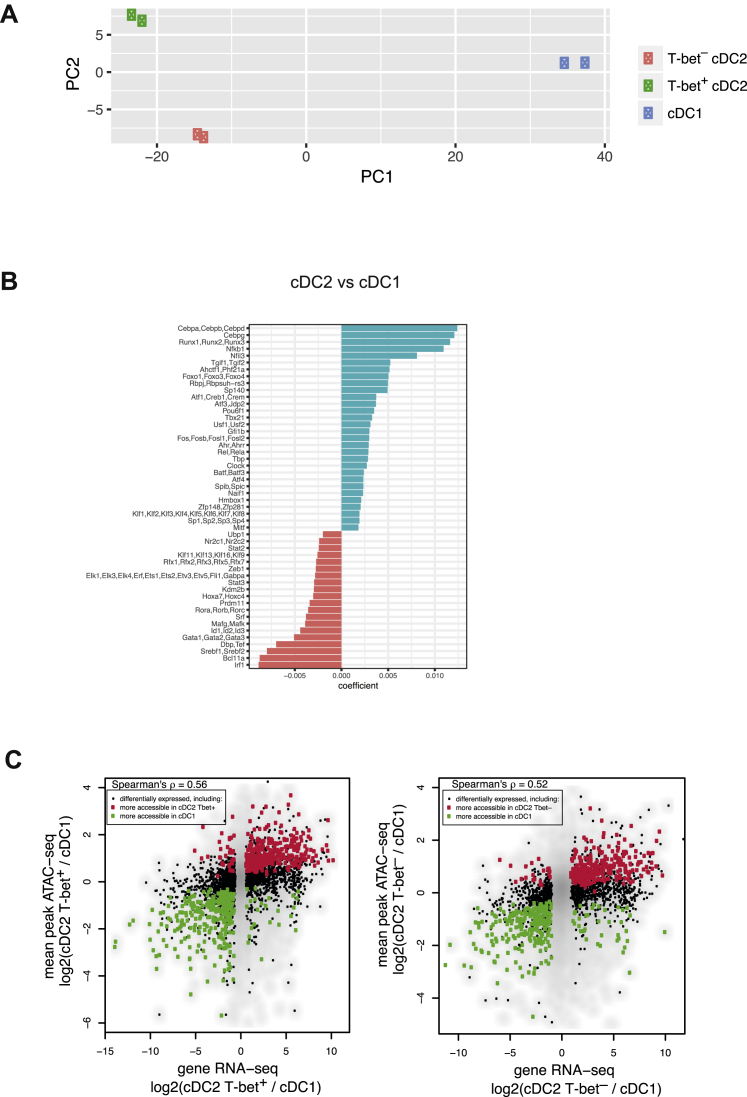
Transcriptional and Epigenetic Landscape of cDCs, Related to Figure 4 (A). Principal component analysis (PCA) for ATAC-seq of cDC1s, T-bet^+^ cDC2s and T-bet^–^ cDC2s. Each symbol represents a biological replicate for each cell-type. (B). Predictive value of TF motifs in peaks more accessible in cDC2s versus cDC1s. (C). Correlation between differential ATAC-seq peak accessibility and gene expression in cDC1s versus cDC2s.

Next, we performed ATAC-seq to assess TF-binding motif enrichment at differentially accessible open chromatin sites in cDC1s, T-bet^+^ and T-bet^–^ cDC2s (Figures 4A and S3A; Table S4). 2,399 peaks (associated with 1,498 genes) were significantly more accessible in T-bet^+^ DCs, and 1,130 peaks (associated with 801 genes) significantly more accessible in T-bet^–^ DCs (Figure 4B).

Consistent with the known role for IRF8 in cDC1 differentiation, an IRF binding motif was the top motif linked to differential chromatin accessibility in cDC1s versus cDC2s (Figure S3B). Similarly, the T-box motif showed the strongest association with increased chromatin accessibility in T-bet^+^ cDC2s (Figure 4C). Besides T-bet, other high-ranking TF motifs in T-bet^+^ cDC2s included Runx, Srebf, and Nr4a, as well as Rbpj, the transcriptional mediator of Notch signaling, suggesting that these TF family members are likely major contributors to regulation of gene expression in T-bet^+^ cDC2s (Figure 4C). Of note, the motif for Nfil3, a TF previously shown to regulate cDC1 development (Kashiwada et al., 2011), was more highly associated with increased chromatin accessibility in cDC2s versus cDC1s (Figure S3B). Although Nfil3 is not required for cDC2 generation, our data suggest a potential role for Nfil3 in regulating some aspects of cDC2 function. In T-bet^–^ DCs, the RORE motif was associated with T-bet^–^ cDC2-specific peaks (Figure 4C), consistent with a role for RORγt^+^ in regulation of gene expression in these cells along with members of CCAAT-enhancer binding protein (C/EBP) and nuclear factor I (NFI) families of TFs. Analysis of TF expression confirmed enrichment of the predicted transcriptional regulators in the respective DC subset, including Nr4a2 and Nr4a3 for T-bet^+^ DCs, and Cebpa and Nfix for T-bet^–^ DCs (Figure 4D). Notably, a number of predicted TFs did not exhibit differential gene expression suggesting post-transcriptional mechanisms regulating their activity.

To link differentially accessible *cis*-regulatory elements to subset-specific gene expression, we assigned ATAC-seq peaks to the nearest genes and observed correlation of differentially accessible peaks with differential gene expression assessed by RNA-seq analysis of cDC subsets (Figures 4E and S3C). Among the genes most upregulated in T-bet^–^ cDC2s were *Rorc* and *S1pr1*, while genes such as *Tbx21*, *Cr2*, and *Cdh17* were downregulated concordantly with a closed state of chromatin at their multiple *cis-*regulatory elements (Figure 4F). Collectively, these data identify distinct sets of transcriptional regulators operational in the newly identified cDC2 subsets. Given the unique transcriptional identity of the two cDC2 subsets, we consider these to be distinct lineages and henceforth refer to these DCs as cDC2A (T-bet^+^) and cDC2B (T-bet^–^).

### Ontogeny of cDC2 Subsets

**Figure S4:**
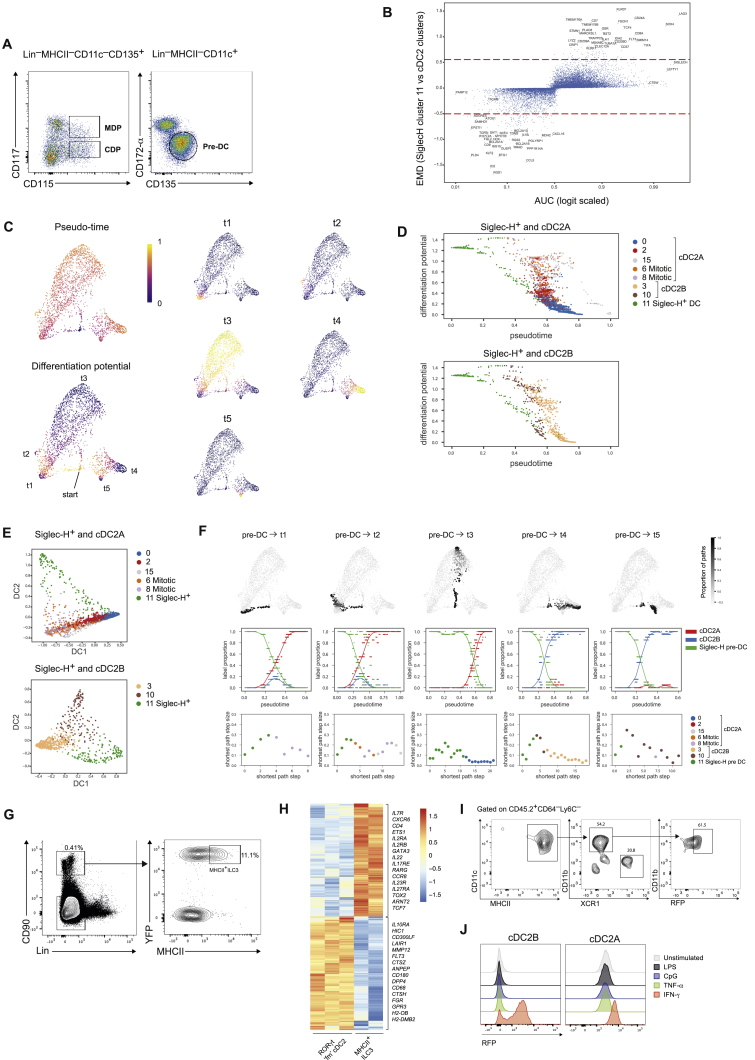
Environmental Cues Drive Distinct DC2 Differentiation Pathways within the Spleen, Related to Figure 5 (A). Gating strategy for the identification of DC progenitors in the bone marrow (BM) (B). Palantir pseudo-time analysis of differentiation potential and branch probabilities from the Siglec-H^+^ pre-DC state to T-bet^+^ cDC2 and T-bet^–^ cDC2 terminal states. (C). Plots showing Palantir differentiation potential (y axis) along Palantir pseudo-time (x axis) for Siglec-H^+^ DC and T-bet^+^ cDC2s (top) or Siglec-H^+^ DC and T-bet^–^ cDC2 clusters (bottom) (D). Plots showing the top two diffusion component embeddings for Siglec-H^+^ DC and T-bet^+^ cDC2 clusters (top) or Siglec-H^+^ DC and Tbet^–^ cDC2 clusters (bottom). Black arrow indicates Siglec-H^+^ DC cluster cells adjacent to cells from the proliferative T-bet^+^ cDC2 clusters 6 and 8. (E). Top panel: plots showing probability of each cell being within 20 nearest neighbors of randomly sampled shortest paths from the Siglec-H^+^ DC to the indicated end points. Middle panel: plots showing the proportion of cells belonging to Siglec-H^+^ DC, T-bet^+^ cDC2, or T-bet^–^ cDC2 from 20 nearest neighbors of randomly sampled shortest paths. Bottom: plots showing diffusion distance step sizes for each step along the indicated shortest paths (bottom panel). Colors illustrate cluster membership. (F). Graph showing AUC (x axis) for genes differentially expressed between Siglec-H^+^ DC cluster (cluster 11) and all other cDC2 clusters. EMD on the y axis. Dashed lines represents μ_EMD_ ± 3σ_EMD_. (G). Gating strategy for FACS-isolation of MHCII^+^ ILC3s: Lin = CD3, CD19, CD49b, Siglec-F. (H). Heatmap reports scaled expression of 3550 differentially expressed genes (log_2_FC > 1, FDR < 0.01) between ILC3s and Rorγt fm cDC2s. Selected genes listed to the right. (I). Representative flow cytometric analysis of phenotypes of splenic progeny from *Tbx21*^*RFP-cre*^ CD45.2^+^Ly6C^−^CD64^–^MHCII^+^CD11c^+^Siglec-H^+^ pre-DCs adoptively transferred into sub-lethally irradiated CD45.1 recipient mice 7 days earlier (data from one experiment with n = 3). J. Sort purified T-bet^+^ or T-bet^–^ cDC2 were cultured for 24hrs in the presence of LPS, CpG, TNF-α or IFN−γ. Representative overlay histogram showing the expression of RFP(T-bet) at 24hrs. Data representative of 2 (TNF-α) or 4 (all other cytokines/TLR agonists) independent experiments, n = 2-3.

**Figure 5:**
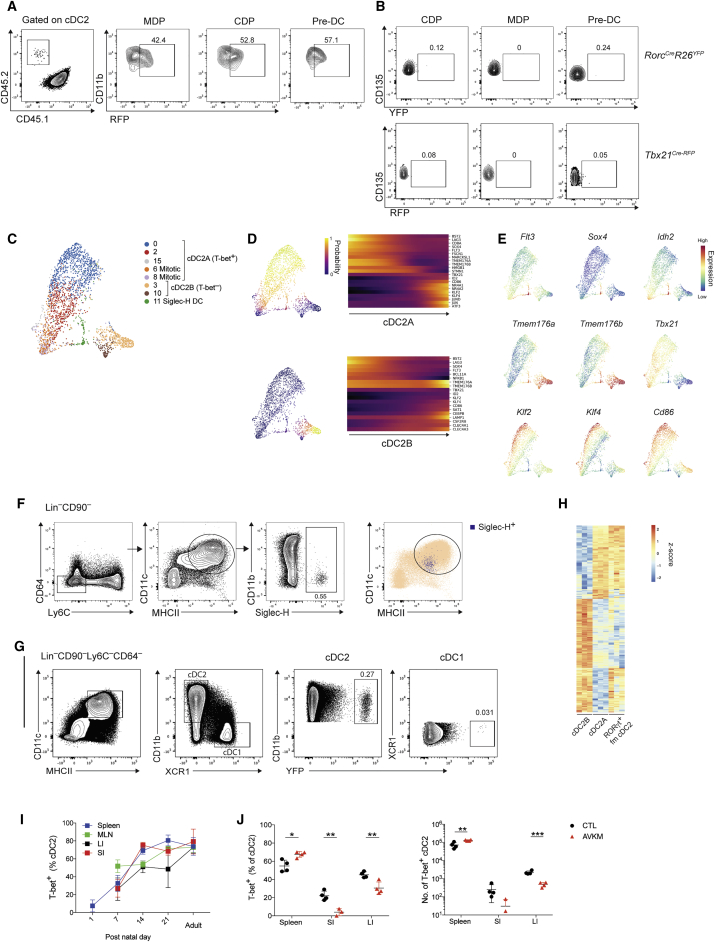
Environmental Cues Drive Distinct DC2 Differentiation Pathways within the Spleen (A) Derivation of splenic CD11b^+^ DC subsets from bone marrow (BM) progenitors. Shown are splenic cDC2s 7 days post transfer. Data representative of MDP and CDP recipients (n = 3) or pre-DC recipients (n = 6) from 3 independent experiments. (B) Analysis of YFP in BM progenitors from *Rorc*^*Cre*^*Rosa26*^*lsl-YFP*^ mice (top) or RFP expression in cells from *Tbx21*^*RFP-cre*^ mice (bottom). (C) t-SNE embedding of diffusion map of T-bet^+^, Tbet^–^ cDC2, and Siglec-H^+^ pre-DC clusters identified in Figure 1E. (D) Palantir branch probabilities from Siglec-H^+^ pre-DC to T-bet^+^ cDC2 (top) or Tbet^–^ cDC2 (bottom) terminal states. Gene expression trends in pseudo-time along corresponding trajectories. (E) Expression of genes identified as varying significantly along the trajectory from the pre-DC cluster to T-bet^+^ or Tbet^–^ cDC2. (F) Expression of Siglec-H in Lin^–^CD90^–^Ly6C^–^CD64^–^CD11c^+^MHCII^+^ cells. Far right: overlay of Siglec-H^+^ DCs against all MHCII^+^CD11c^+^ DCs. (G) Representative plot showing percentage of YFP^+^ cells in splenic cDC1 and cDC2 populations in *Rorc*^*Cre*^*Rosa26*^*lsl-YFP*^ mice. (H) Heatmap showing expression of genes differentially expressed between bulk T-bet^+^ and Tbet^–^ cDC2s across T-bet^+^, Tbet^–^, and Rorγt fate-mapped (fm) cDC2s. (I) Frequency of T-bet^+^ cDC2s within the spleen, MLN, large intestine lamina propria (LI), and small intestine lamina propria (SI) at indicated time point post birth. n = 3 per time point, error bars represent mean ± SEM. For the day 7 analysis SI and LI tissues were pooled from 2 mice per sample. (J) Generation of T-bet^+^ cDC2s in mice treated with a broad-spectrum antibiotic cocktail (AVKM) or H_2_O (control). Each symbol represents one mouse. Error bars represent mean ± SEM. See also Figure S4 and Table S5.

DCs arise from myeloid progenitors that differentiate first in the bone marrow (BM) and then in the spleen. To determine where and at what stage of differentiation the divergence of cDC2s into the two identified subsets occurs, we isolated macrophage and DC progenitors (MDP), common DC progenitors (CDP), and pre-DCs from the BM of *Tbx21*^*RFP-Cre*^ mice (Figure S4A) and transferred them into CD45.1 congenic mice. Analysis of splenic DCs 7 days after transfer demonstrated that MDP, CDP and pre-DCs could give rise to both cDC2A (T-bet^+^) and cDC2B (T-bet^–^) (Figure 5A) indicating that both cDC2 subsets arise relatively late within the same developmental pathway. Thus, even though some monocyte associated genes were enriched in cDC2B, these cells appear to arise from classical DCs consistent with their expression of *Zbtb46* (Figure 3A). Single-cell analysis of DC progenitors in the BM has identified immediate precursors poised for either cDC1 or cDC2 cell fate (Grajales-Reyes et al., 2015, Schlitzer et al., 2015) raising the possibility that cDC2A and cDC2B emerge during differentiation in the BM. However, our analysis of BM progenitors for expression of T-bet or RORγt in *Tbx21*^*RFP-Cre*^ or *Rorc*^Cre^-*Rosa26*^*lsl-YFP*^ mice, respectively, failed to reveal any progenitor cells expressing T-bet (RFP) or RORγt (YFP) at any stage of BM differentiation (Figure 5B). This suggests that cDC2s acquire specific transcriptional profiles in the periphery, likely in response to environmental cues.

To gain further insights into developmental relationships between cDC2 subsets, we applied the Palantir algorithm to model DC fate commitment using pseudo-time (Setty et al., 2018). Consistent with a progenitor phenotype, cells in the putative Siglec-H^+^ pre-DC population (cluster 11; Figure 1E) were distinguished by high levels of *Flt3* (Figure S4B), known to regulate DC differentiation (McKenna et al., 2000), and by genes related to cell division (*Stmn1* and *Tuba1a*; Figure S4B). Therefore, we specified the starting point for Palantir within this pre-DC population to characterize potential differentiation trajectories from the Siglec-H^+^ cells toward cDC2A and cDC2B. Given that cell-cycle signatures can confound differentiation trajectories, we employed factor analysis to remove cell-cycle effects before applying Palantir (Buettner et al., 2017). The results were visualized using t-SNE embedding of diffusion components (Figure 5C).

From the Siglec-H^+^ cluster starting point, Palantir determined terminal states in both T-bet^+^ and T-bet^–^ clusters (Figures 5D and S4B). The differentiation potential rapidly diminished upon exit from the Siglec-H^+^ cluster for both cDC2A and cDC2B clusters (Figure S4C) with robustness analysis supporting the possibility of both cDC2 subsets arising from a common splenic Siglec-H^+^ DC progenitor (Figures S4D and S4E). Examination of transcripts varying along these trajectories revealed the successive downregulation of Bst2, Lag*3*, Sox4, Flt3, Tmem176a, and Tmem176b along with upregulation of Tbx21, Id2, CD86, Klf2, and Klf4 *(*Figures 5D and 5E). Cells that differentiated into cDC2B retained expression of Tmem176a and Tmem176b and upregulated CD86 and Klf4 (Figures 5D and 5E) suggesting an increasing state of maturity. Together, these analyses highlight cDC2 differentiation paths within the spleen and define genes associated with cDC2 specification.

Although the pre-DC population shared a number of genes with cDC2s, they could be distinguished based on the absence of Itgam (CD11b) expression (Figure 1E) and by unique expression of *Siglech* (Figure S4F). Flow cytometric analysis validated the presence of these precursor cells in the spleen (Figure 5F). Siglec-H^+^ pre-DCs were enriched in CD11c^int^MHCII^int^ cells (Figure 5F), indicative of an immature DC phenotype. Tmem176a and Tmem176b were highly expressed by cells within the Siglec-H^+^ cluster (Figures 5E and S4F) suggesting that RORγt expression was acquired prior to their differentiation into mature cDC2 subsets. Given the absence of Rorc expression at the pre-DC stage (Figure 5A), we reasoned that the expression of Rorc by the putative Siglec-H^+^ precursor would allow us to follow their developmental trajectory using genetic fate-mapping of this population in *Rorc*^*Cre*^*Rosa26*^*lsl-YFP*^ mice. This analysis revealed the presence of YFP^+^ cells in both cDC1 and cDC2 subsets (Figure 5G). Furthermore, RNA-seq analysis of sorted YFP^+^ cDC2s demonstrated a gene signature encompassing both cDC2A and cDC2B defining genes (Figure 5H) suggesting that at least some of the cells in both of these subsets can originate from a RORγt^+^ precursor. MHCII expression has been described in a subset of ILC3s (Gury-BenAri et al., 2016, Hepworth et al., 2013). To confirm that RORγt-expressing DCs were distinct from MHCII^+^ ILC3s, we compared the transcriptomes of RORγt fate-mapped cDC2s to those of splenic MHCII^+^ ILC3s (Figure S4G). As expected, genes upregulated in MHCII^+^ ILC3s included signature ILC3 genes (*Il7r*, *Il23r*, *Cxcr6*, *Tox*, *Ets1*) (Figure S4H). Conversely, RORγt fate-mapped cDC2s expressed canonical DC genes (*Dpp4* and *Flt3*) (Figure S4H). Last, FACS sorted Siglec-H^+^ DCs transferred into congenically marked recipient mice gave rise to both splenic cDC2 populations (Figure S4I), confirming their progenitor nature.

The frequency of cDC2A varied across peripheral lymphoid and non-lymphoid tissues (Figure 1B). cDC2As were enriched at mucosal barrier sites (Figure 1B), indicating a potential role for the microbiota in driving T-bet expression. Accordingly, we observed dramatic increases in the frequency of intestinal cDC2A between birth and weaning, a period associated with increased microbial colonization (Figure 5I). To assess the impact of microbiota on cDC2 composition, we administered a cocktail of broad-spectrum antibiotics to pregnant females and analyzed cDC2 phenotypes in adult offspring maintained on antibiotics. Antibiotic treatment resulted in reduced frequencies and numbers of cDC2A within the intestine but not the spleen compared to age-matched untreated mice (Figure 5J). This suggests that microbiota dependent signals promote T-bet expression at least in some DCs. A recent study reported induction of T-bet expression in mediastinal LN DCs in response to tumor necrosis factor alpha (TNF-α) or lipopolysaccharides (LPS) stimulation (Bachus et al., 2019). While we also observed increased frequencies of T-bet^+^ cDC2As following infection with pathogens known to induce type 1 inflammation (data not shown), we found that IFN-γ, but not TNF-α or TLR signaling, promotes T-bet expression in DC (Figure S4J). Overall, these results suggest that signals within the tissue microenvironment direct cDC2 differentiation toward T-bet^+^ cDC2A.

### cDC2A and cDC2B Have Distinct Phenotypic and Functional Properties

**Figure 6:**
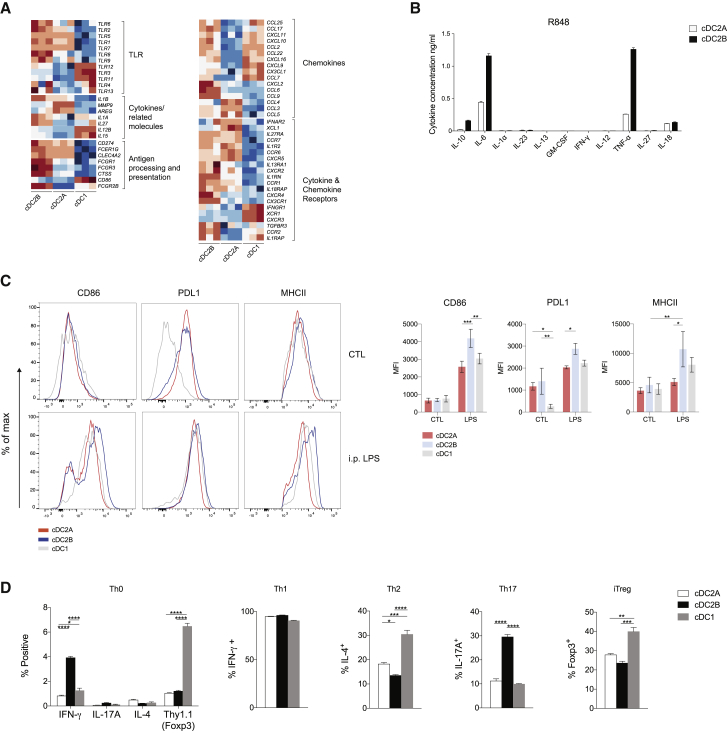
T-bet^+^ and T-bet^–^ cDC2s Are Phenotypically and Functionally Distinct (A) Heatmap of select TLRs, chemokines, cytokines, and their receptors for genes differentially expressed between bulk cDC1s, T-bet^+^, and T-bet^–^ cDC2s. (B) Cytokines detected in culture supernatant 18 h after stimulation with R848, analyzed using a multiplexed cytokine assay. Data are mean ± SEM from triplicate culture wells. (C) Cell surface expression of CD86, PDL1, and MHC class II on indicated DC subset 16 h after intraperitoneal (i.p.) immunization with LPS. Left: representative overlay histogram; right: composite bar graphs of median fluorescence intensity (MFI) (n = 3). (D) Sorted naive OTII CD4^+^ T cells were cultured with indicated DC subset and OVA peptide under non-polarizing (Th0) or polarizing conditions for 4 days. Bar graphs of summary data for intracellular cytokine production following restimulation or expression of Thy1.1(Foxp3) in unstimulated cells. Data shown as mean ± SEM; p values were calculated using two-way ANOVA (Th0) or one way ANOVA. See also Figure S5.

To assess potential functional differences between cDC2 subsets, we examined differential expression of genes encoding immune modulatory and signaling molecules including TLRs, cytokines, chemokines, chemokine receptors, and molecules related to antigen processing and presentation. The two cDC2 subsets markedly varied from one another and from cDC1s in expression of TLR genes: cDC1s exclusively expressed TLR3, TLR11, and TLR12, whereas cDC2B preferentially expressed TLR1, TLR2, TLR5, TLR6, TLR7, TLR8, and TLR9 (Figure 6A). The expression of most of these TLRs was notably absent or reduced in cDC2A with the exception of TLR1, TLR5, and TLR7 (Figure 6A). Thus, the identified cDC2 subsets are capable of sensing and responding to distinct sets of innate immune stimuli. Furthermore, genes encoding chemokines and chemokine receptors, as well as cytokines receptors, also exhibited distinct expression patterns across the three cDC subsets (Figure 6A). cDC2B exclusively expressed *Cxcl2*, *Ccl6*, and *Ccl9*, whereas cDC2A had increased expression of *Ccl3*, *Ccl4*, and *Ccl5* (Figure 6A). While the expression of several other chemokines was shared between cDC2B and cDC1s, they were notably absent from cDC2A (Figure 6A). The unique repertoires of chemokines and chemokine receptors expressed by the two cDC2 subsets indicate divergent functions *in vivo*.

**Figure S5:**
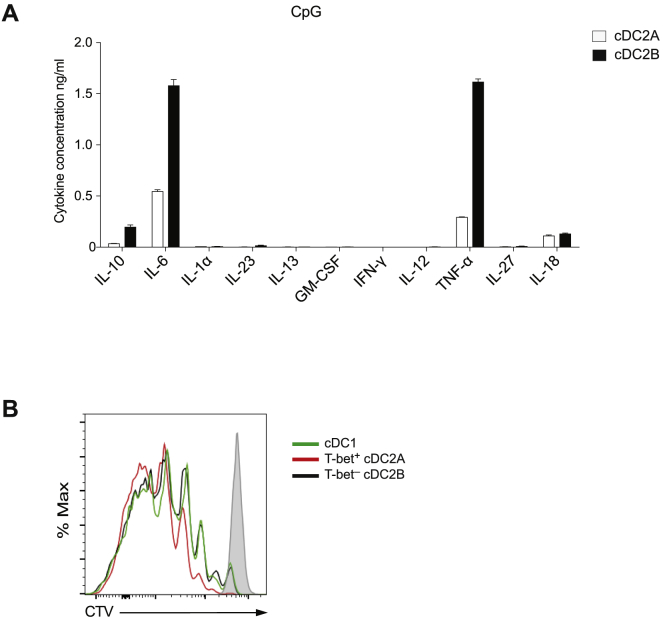
T-bet^+^ and T-bet^–^ cDC2s Are Functionally Distinct, Related to Figure 6 (A). Cytokines detected in culture supernatant 18 hours after stimulation with CpG using a multiplexed cytokine assay. (B). Proliferation of naive OTII CD4^+^ T cells 5 days after co-culture with OVA peptide and either T-bet^+^ cDC2s, T-bet^–^ cDC2s or cDC1s. Data, shown as mean ± SEM, are representative of 2 independent experiments (n = 3).

The current view holds that cDC1s initiate CD8 T cell responses while cDC2s prime CD4 T cells (Dudziak et al., 2007, Hildner et al., 2008). To determine if the cDC2 lineages have differential abilities to polarize CD4 T cells, we first compared cytokine gene expression across the cDC subsets. cDC2B had significantly higher expression of genes encoding IL-1α and IL-27 (Figure 6A), whereas cDC2A expressed higher levels of transcripts associated with tissue repair such as amphiregulin (Areg) and the metalloproteinase MMP-9 (Figure 6A). MMP9 has been shown to modulate cytokine activity through activation of TGF-β (Yu and Stamenkovic, 2000) and via inhibition of IL-23 expression (Oriss et al., 2014). To further explore the pro- and anti-inflammatory potential of cDC2 subsets, we analyzed their responses to R848, an agonist for TLR7 whose expression was similar in both cDC2 subsets. cDC2A secreted significantly less TNF-α and IL-6 than their cDC2B counterparts suggesting a markedly lower pro-inflammatory potential of the former (Figure 6B). We also observed reduced levels of pro-inflammatory cytokines by cDC2A upon stimulation with CpG (Figure S5A), further highlighting functional differences between cDC2 subsets. In the steady state, both cDC2 subsets displayed equivalent levels of MHC class II, co-stimulatory molecules, and PDL1. However, cDC2B expressed higher levels of co-stimulatory molecules and MHC class II upon *in vivo* LPS challenge (Figure 6C).

We next isolated cDC2A and cDC2B and co-cultured them with naive OTII CD4^+^ T cells along with their cognate ligand, OVA_323–339_, either in the absence (“Th0”) or presence of polarizing cytokines. Consistent with the markedly diminished pro-inflammatory responses to TLR agonists, cDC2A had reduced ability to polarize naive T cells toward IFN-γ or IL-17A producing T cells under Th0 conditions (Figure 6D), despite similar ability to induce T cell proliferation (Figure S5B). Diminished IL-17 production was also observed under Th17 polarizing conditions (Figure 6D). Both cDC2 subsets exhibited a similar capacity to support Foxp3^+^ T cell differentiation *in vitro* (Figure 6C). Thus, cDC2A and cDC2B are phenotypically and functionally distinct and are likely to have divergent anti- and pro-inflammatory roles *in vivo*.

### Identification of Novel cDC2 Subsets in Humans

**Figure 7:**
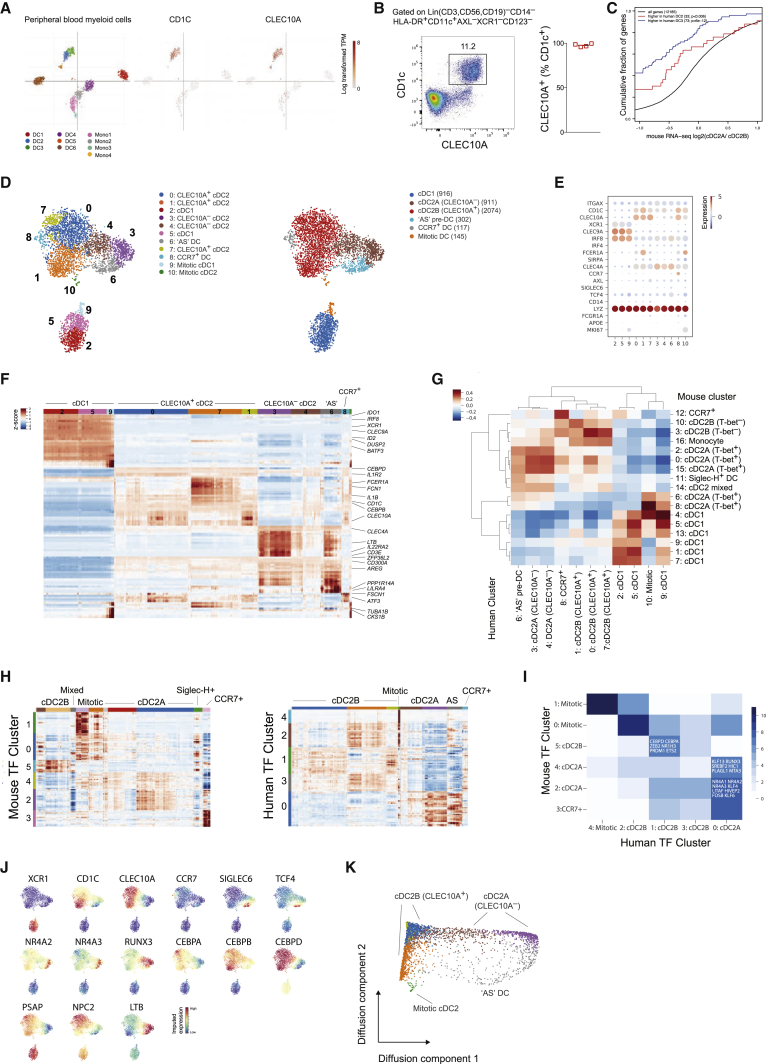
Conservation of DC Subsets across Species (A) t-SNE map of human peripheral blood DCs from Villani et al. (2017), colored by cell type or log-transformed expression of labeled genes (middle and right panel). (B) Expression of CD1c and CLEC10A within peripheral blood cDC2s. Bar graph shows summary frequencies for four individual donors. (C) Distribution of expression changes between T-bet^+^ and T-bet^–^ cDC2s. Genes up- or downregulated in human “DC2” versus “DC3” cDC2s (Villani et al., 2017) are shown. (D) t-SNE map of 4,465 human splenic DCs. Colors indicate unsupervised clustering by Phenograph (left) or classification based on expression of canonical markers (right). Each dot represents an individual cell. (E) Expression of canonical myeloid genes across the transcriptionally defined DC clusters from (D). (F) Heatmap reporting scaled, imputed expression for the top differentially expressed genes for each cluster identified in (D). (G) Pearson correlation between human and mouse spleen scRNA-seq DC clusters. Mouse spleen DC clusters from Figure 1E. (H) Heatmap reporting scaled, imputed expression for the 103 top varying TF genes across cDC2s in each species. Rows are ordered by the TF gene cluster determined using Phenograph and columns are ordered by the annotated cDC2 cluster. (I) Heatmap reporting overlap of top varying TF genes for mouse and human TF gene clusters identified in (H). Each colored count indicates the number of TFs that belong to both the mouse (rows) and human TF gene cluster (columns) indicated. TF gene clusters are further annotated with the cDC2 subset concordant with their expression profile in (H). (J) t-SNE map of human splenic DCs colored by imputed expression of canonical human DC genes (top) or cDC2 lineage-defining TF and marker genes (middle and bottom) identified in Figures 2A and 3D. (K) Diffusion component analysis of human spleen DC clusters identified in (D) illustrating gradients from “AS” DCs to cDC2A and cDC2B clusters. See also Figure S6 and Tables S5, S6, and S7.

**Figure S6:**
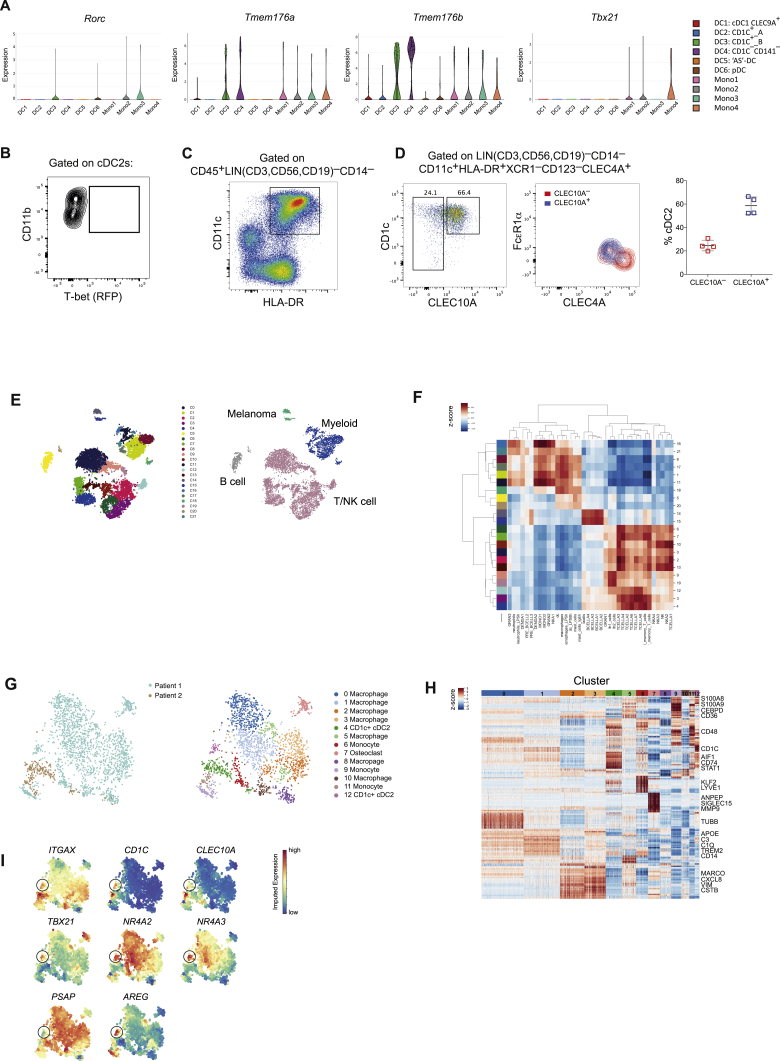
Human DC Heterogeneity, Related to Figure 7 (A). Violin plots showing expression distribution of mouse DC subset marker genes across human peripheral blood DC and monocyte clusters identified in Villani et al. (2017). (B). Representative flow cytometric analysis of mouse peripheral blood cDC2s showing absence of T-bet (RFP)^+^ cDC2s. (C). Gating strategy for FACS-isolation of human spleen DCs for scRNA-seq. DCs were defined as live, LIN(CD3,CD56,CD19)^−^CD14^–^CD11C^+^HLA-DR^+^. (D). Representative flow cytometry analysis of human spleen cDC2s gated as Lin(CD3,CD56,CD19)^–^CD14^–^CD11c^+^HLA-DR^+^CD123^–^XCR1^–^CLEC4A^+^ cells. Left panel: cell surface expression of CD1c and CLEC10A by cDC2s. Right panel: overlay of CLEC10A^+^ and CLEC10A^–^ cDC2s distinguished by differential expression of CLEC4A and FcεR1a. Summary bar graphs show frequency of CD1C^+^CLEC10A^+^ and CD1C^+^CLEC10A^–^ cDC2s as a percentage of cDC2s (n = 4 individuals). (E). *t*-SNE embedding of 9,315 FACS-isolated CD45^+^ immune cells from two melanoma tumors. Colors indicate unsupervised clustering by Phenograph (left panel) or classification based on expression of canonical markers and correlations with bulk RNA-seq data (right panel). Each dot represents an individual cell. (F). Pearson correlations between cluster centroids in (F) and bulk RNA-seq data from purified immune populations (Jeffrey et al., 2006, Novershtern et al., 2011) (G). t-SNE map of 2,122 myeloid cells identified in (F). Colors indicate patient sample (left) or unsupervised clustering by Phenograph (right panel). Each dot represents an individual cell. (H). Heatmap of normalized, log transformed and MAGIC imputed expression of top 20 differentially expressed genes, defined by the highest earth mover’s distance (EMD), per Phenograph cluster in E. The colored bar at the top of the heatmap shows assignment of cells to clusters labeled in F, right panel. (I). t-SNE map of human melanoma myeloid cells (H) colored by imputed expression of labeled genes.

Similar to mouse cDC1s, human cDC1s express XCR1 and CLEC9A whereas cDC2s are defined by CD1c expression (Crozat et al., 2010, Dzionek et al., 2000, Huysamen et al., 2008). Comparative transcriptomics and functional studies have demonstrated correspondence between mouse and human cDC subsets (Haniffa et al., 2012, Robbins et al., 2008). To determine if counterparts of the newly characterized mouse cDC2 subsets exist in human blood we examined the correlation between gene expression characteristic of the identified mouse DC clusters and gene signatures of human DCs extracted from a recently published scRNA-seq analysis of human peripheral blood myeloid cells (PBMCs) (Villani et al., 2017). In the latter study, two subsets of human cDC2s were identified (dubbed “DC2” and “DC3”). Consistent with previous studies demonstrating that CLEC10A is expressed by all human CD1c^+^ cDC2s (Heidkamp et al., 2016, Heger et al., 2018), both “DC2” and “DC3” clusters expressed CLEC10A (Figure 7A), indicating that CD1c^+^ cDC2s may be analogous to the CLEC10A^+^ cDC2B subset identified in mice. Flow cytometric analysis of PBMCs from 4 independent donors confirmed that blood CD1c^+^ DCs are uniformly CLEC10A^+^ (Figure 7B). To determine the relationship between mouse and human cDC2 subsets, we compared the similarity of orthologous signature gene expression for each DC cluster. We found that the previously reported division in human blood cDC2 subsets did not align with the cDC2 division observed in mice (Figure 7C). Instead, cDC2B genes were upregulated in both previously reported human peripheral blood cDC2 subsets (Figure 7C). Tmem176a- and Tmem176b-expressing cells were present at high frequency and *Rorc* transcript was detectable, albeit at low frequency, in human “DC3” cells (Figure S6A). Collectively these data indicate that human peripheral blood CD1c^+^CLEC10A^+^ cDC2s are analogous to murine cDC2B cells. The lack of human cDC2A in the peripheral blood was consistent with the absence of the corresponding subset in mouse PBMCs (Figure S6B).

To identify human counterparts to the mouse cDC2A subset, we undertook a scRNA-seq analysis of Lin(CD3,CD56,CD19)^–^CD14^–^CD11c^+^HLA-DR^+^ DCs isolated from human spleen (Figure S6C). Clustering of 4,465 single-cell transcriptomes using Phenograph identified 11 clusters (Figure 7D). Analysis of canonical human myeloid genes (Figure 7E) revealed three clusters encompassing CLEC9A^+^XCR1^+^ cDC1 cells, one cluster distinguished by high levels of CCR7 expression, a cluster corresponding to the recently characterized AXL^+^SIGLEC6^+^ (“AS”) pre-DCs, 2 clusters of mitotic DCs, and 5 remaining clusters classified as cDC2s based on their expression of IRF4 and CLEC4A (DCIR) (Bajaña et al., 2016, Vu Manh et al., 2015). Of the cDC2 clusters, 2 contained the canonical CD1c^+^CLEC10A^+^ subset while the remaining 2 clusters (3 and 4) expressed lower levels of CD1c transcript and lacked CLEC10A (Figure 7E). Flow cytometric analysis of DCs from the original sample and three additional spleen samples confirmed the existence of two major distinct populations of human CD1c^+^ cDC2s, delineated by CLEC10A expression (Figure S6D). In agreement with the scRNA-seq analysis (Figure 7E), CLEC10A^+^ cDC2 cells were distinguished by increased cell-surface expression of FcεR1α and reduced expression of CLEC4A compared to their CLEC10A^–^ counterparts (Figure S6E). These data suggest that CD1c^+^CLEC10A^+^CLEC4A^lo^ cDC2 and CD1c^lo^CLEC10A^–^CLEC4A^hi^ cDC2s are human counterparts of cDC2B and cDC2A, respectively.

Analysis of genes differentially expressed between DC clusters showed that gene signatures of human cDC2A and cDC2B subsets included mouse cDC2 subset signature gene (Figure 6F). Similar to the division in mouse cDC2s, human cDC2B exhibited a more pro-inflammatory phenotype with increased expression of IL1B, whereas human cDC2A expressed higher levels of transcript for amphiregulin (AREG), IDO1, the immunomodulatory receptor CD300a, and IL22 binding protein (IL22RA2) (Figure 7F). To determine the overall correspondence between mouse and human DC subsets, we compared the similarity of orthologous genes signatures for each splenic DC cluster. This confirmed that human CLEC10A^+^ cDC2s aligned with mouse cDC2B, whereas CLEC10A^–^ cDC2s showed a greater degree of correspondence with mouse cDC2A (Figure 7G). We reasoned that if overall transcriptional features of cDC2 subsets were conserved across species, transcriptional regulators and genes reflecting functional specialization would also be conserved. Indeed, unsupervised clustering of TF gene expression profiles in mouse and human cDC2s confirmed the transcriptional basis for division of cDC2A and cDC2B in both species (Figure 7H) and demonstrated an overlap between the defining mouse and human TFs for each cDC2 subset (Figure 7I). cDC2 transcriptional regulators identified in mice showed clear concordant patterns of expression in human cDC2 subsets with differential expression of RUNX3, NR4A2, NR4A3, SREBF2, and CEBP family member transcripts delineating CLEC10A^+^ and CLEC10A^–^cDC2s (Figure 7J). In addition to TFs, genes associated with lipid antigen presentation and metabolism (CD1E, NPC2, PSAP) were enriched in CLEC10A^+^ cDC2B (Figures 7F and 7J), analogous to mouse cDC2B. Human cDC2A cells were notable for their expression of CD3E (Figure 7F). Notch signaling has been shown to induce intracellular expression of CD3ε in human natural killer (NK) cells (De Smedt et al., 2007), suggesting that Notch signaling may be a conserved feature between mouse and human cDC2A. Together, these findings indicate that functional division between cDC2A and cDC2B is likely conserved.

Human and mouse CCR7^+^ DC clusters also exhibited a similar transcriptional signature (Figure 7G), indicating a conserved phenotype. AS DCs were distinguished by high expression of *PPP1R14A* and *DAB2*, signature genes previously defined in their peripheral blood counterparts (Villani et al., 2017; Figure 7F). In addition, AS DCs resembled the murine Siglec-H^+^ pre-DC, confirming the existence of a splenic progenitor DC with overlapping features of pDCs and cDC2s in both species. As with the murine cDC2s, we employed diffusion maps to assess the relationships between the AS DC and human cDC2 subsets identified. This analysis revealed branches connecting the AS pre-DC cluster to both cDC2 subsets, in particular the mitotic cDC2 cluster (Figure 7K). Enrichment of Wnt signaling pathways in AS DCs (Table S5) observed by GSEA suggests an important role for Wnt in DC differentiation.

**Figure S7:**
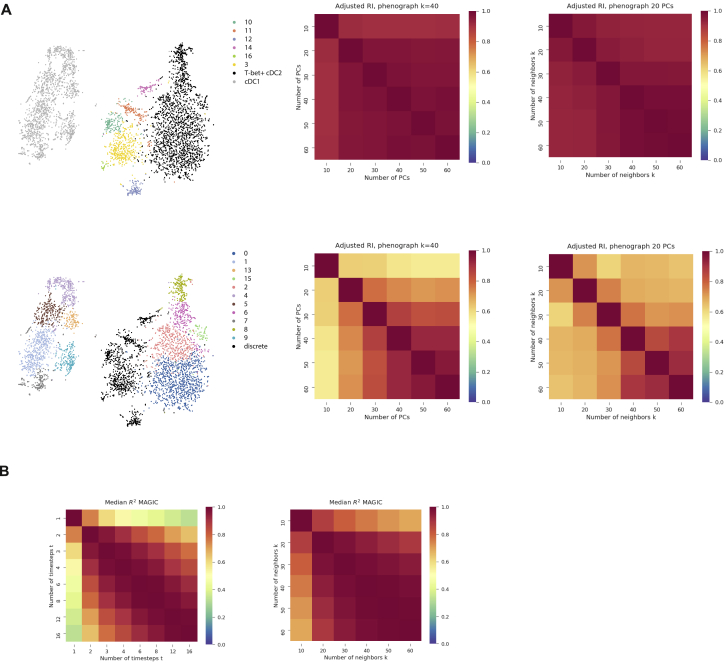
Identification of cDC2 Heterogeneity, Related to Figures 1 and 2 and STAR Methods (A). Clustering robustness measured by adjusted Rand Index (RI) for ranges of principal components and k supplied to Phenograph. In the top panel, with cDC1 (gray) and T-bet+ cDC2 (black) clusters consolidated, RI values indicate robust partitioning between the remaining clusters. In bottom row, when all other cDC2 clusters are consolidated (black), RI values indicate that partitioning is not reliable within cDC1 and T-bet+ cDC2. (B). Heatmap showing the median Rˆ2 value across all genes for MAGIC imputed values calculated with varying timestep (left panel) and number of neighbors (right panel).

We reasoned that the observed pro-inflammatory properties of mouse T-bet^–^ cDC2s might suggest their role in tissue inflammation whereas the markedly diminished inflammatory potential of T-bet^+^ cDC2s suggests that this subset might be enriched in environment with prominent tissue remodeling and repair and immunomodulatory features such as tumors. To assess human cDC2 heterogeneity and specifically the presence of cDC2A within the tumor microenvironment, we performed scRNA-seq analysis of immune cells from human melanoma. We analyzed FACS-isolated CD45^+^ cells from two patients (Table S6) and identified 2,122 myeloid cells (Figures S6E and S6F). Re-clustering of the myeloid cells to achieve greater resolution identified 13 clusters comprising monocytes, macrophages, pDCs, and cDC2s (Figures S6G – S6I). CD1c^+^ cDC2s (clusters 4 and 12) were patient-specific (Figure S7G) with *TBX21*-expressing cells identified within a patient-specific CD1c^+^ cluster (cluster 12; Figure S6I). Genes significantly upregulated in this cluster included signature cDC2A genes such as *AREG* and *NR4A3* (Figure S6; Table S7). Collectively, these analyses confirm that cDC2A (T-bet^+^) and cDC2B (T-bet^−^) counterparts are present within human tissue and share key transcriptional regulators and phenotypes with the corresponding mouse cDC2 lineages.

## Discussion

In this study, we analyzed the transcriptional landscape and heterogeneity of murine DCs. We found that mouse cDC2s, in semblance of major ILC or CD4 T cell subsets, can be broadly divided into two subsets, delineated by T-bet (cDC2A) or its absence (cDC2B). The latter cDC2B subset expressed RORγt along with C/EBP family members. Analysis of defining gene signatures for each cDC2 subset suggests a high degree of functional specialization within cDC2s and may be indicative of division of labor between cDC2A and cDC2B. We extended these findings to human splenic DCs by demonstrating a similar sub-division among human CD1c^+^ cDC2s through identification of conserved transcriptional regulators and distinguishing marker genes. Of note, human blood harbors only the cDC2B population, highlighting the importance of studying human DCs within lymphoid tissues.

Although potential heterogeneity within the cDC2 population has been suggested by differential expression of cell surface markers, such as Esam and Mgl2 (Kumamoto et al., 2009, Lewis et al., 2011), these markers vary across tissues and it was unclear if cDC2s identified by these markers represent separate cDC2 subsets. Our study revealed the existence of distinct cDC2 lineages, their transcriptional identity and developmental origins. We show that splenic cDC2s previously identified by cell surface expression of Esam largely belong to the cDC2A subset. In contrast to Esam^+^ cells that have only been identified in the spleen and MLN (Lewis et al., 2011, Satpathy et al., 2013), we find T-bet^+^ cDC2A present throughout lymphoid and non-lymphoid tissue. T-bet^–^ cDC2B encompass Mgl2^+^ DCs, previously identified in the spleen and skin draining LNs, and are the predominant cDC2 subset in a number of tissues. The finding that RORγt expression is associated with a major subset of cDC2s is notable because it was thought that RORγt expression was restricted to thymocytes, Th17 cells, and ILC3s (Eberl et al., 2004). Furthermore, MHC class II gene ablation in *Rorc*-expressing cells has been used to assess antigen-presenting function of ILC3s (Hepworth et al., 2013, Hepworth et al., 2015, Melo-Gonzalez et al., 2019), which was proposed to be important for mucosal T cell tolerance and Tfh differentiation (Hepworth et al., 2013, Melo-Gonzalez et al., 2019). A possible contribution of MHC class II loss by RORγt-expressing cDC2B or Siglec-H^+^ pre-DC to these effects needs to be considered.

The distinguishing features of cDC2 subsets identified provide a framework for future exploration of their development and function in steady state and inflammation. In addition to T-bet and RORγt, *in silico* modeling suggested that several other TFs with differential activity in cDC2A versus cDC2B subsets are likely working in consort to generate their unique gene expression patterns. Runx, a known binding partner for T-bet in T cells (Djuretic et al., 2007, Lazarevic et al., 2011), was highly predictive of increased chromatin accessibility in cDC2A, along with Srebf2. Although Srebf2 was not differentially expressed between mouse cDC2 subsets, changes in intracellular pH can target translocation of Srebf2 to the nucleus (Kondo et al., 2017), which may link observed differences in metabolic profiles of cDC2s to their transcriptional outputs.

In addition to delineating cDC2 heterogeneity, we identified additional DC subsets including a population of CCR7^hi^ DCs within CD11b^–^XCR1^–^ cells that transcriptionally aligned with MHCII^hi^ “migratory” DCs isolated from skin-draining LNs. These cells have not previously been observed within the spleen. In addition, we identified a splenic Siglec-H^+^ DC progenitor, comprising ∼0.4% of CD11c^+^MHCII^+^ cells, with a mixed cDC2-pDC phenotype akin to CD11c^+^HLA-DR^+^CD123^+^ pre-DCs recently described in separate single-cell analyses of human DCs (See et al., 2017, Villani et al., 2017). Siglec-H^+^ DC progenitors with pDC and cDC potential have previously been identified within the bone marrow (Schlitzer et al., 2015). A recent study showed that expression of BM Siglec-H^+^Ly6C^–^ pre-DCs expressing Bst2 and Tcf4, analogous to the pre-DCs identified in our dataset, were committed to the pDC lineage (Dress et al., 2019). However, our data would suggest that these progenitors retain cDC potential. Further studies are required to assess the significance of this DC differentiation pathway as well as the relationship between BM-derived pre-DCs and the splenic Siglec-H^+^ precursor.

Our findings that bone marrow DC progenitors lack expression of T-bet and RORγt suggest that cDC2s acquire expression of the respective TFs in response to environmental cues. Indeed, perturbation of the commensal microbiota upon chronic antibiotic treatment led to alterations in the frequency of T-bet^+^ cDC2s within the affected tissues. Although these findings were seemingly in line with a recent study, in which increased TNF-α levels observed in allergic Th2 lung inflammation were proposed to influence the induction of T-bet expression in cDC2s in mediastinal LNs (Bachus et al., 2019), we observed T-bet induction in DCs in response to IFN-γ, but not TNF-α or TLR stimulation by LPS or CpG consistent with STAT1-dependence of T-bet expression (Lighvani et al., 2001).

Our studies suggest that the newly defined cDC2s likely have distinct roles in the recruitment and activation of immune effector cells. Both mouse and human cDC2A were distinguished from cDC2B by expression of Areg, a molecule linked to tissue repair, raising the possibility of an important role in cDC2 function. One of the main challenges of ascribing distinct functional roles to cDC2 subsets has been the lack of DC-specific markers that could be used to deplete cDC2 subsets. Previous efforts to determine functional roles of cDC2s have relied on the use of CD11c transgene encoded Cre recombinase to target cDC2 subsets through the deletion of IRF4, Klf4, or components of the Notch signaling pathway (Gao et al., 2013, Lewis et al., 2011, Satpathy et al., 2013, Schlitzer et al., 2013). One caveat to these experiments is the expression of CD11c by non-DCs including macrophages, NK cells, T cells, and plasma cells (Caton et al., 2007, Hebel et al., 2006, Jung et al., 2002, van Rijt et al., 2005). Furthermore, we found that neither IRF4 nor Klf4 expression distinguishes the cDC2 subsets identified. The distinct expression of T-bet, RORγt, along with other discriminatory TFs in particular subsets of DCs will allow the development of novel genetic means of targeting cDC2 subsets with increased precision and further elucidation of their function in tissue homeostasis and immune regulation. The correspondence between the newly defined cDC2 subsets across species provides a framework for the translation of mouse dendritic cell studies into knowledge of their functions in human health and disease.

## STAR★Methods

### Key Resources Table

**Table:** 

REAGENT or RESOURCE	SOURCE	IDENTIFIER
**Antibodies**		

Anti-mouse CD86 (AlexaFluor700)	ThermoFisher	Cat#17-0862-82; RRID: AB_469419; Clone: GL-1
Anti-mouse PDL1 (PE-Cy7)	BioLegend	Cat#124313; RRID: AB_10639934; Clone: 10F.9G2
Anti-mouse CD115 (APC)	Tonbo Bioscience	Cat#20-1152; RRID:AB_2621586; Clone AFS98
Anti-mouse CD117 (APC-eFluor 780)	ThermoFisher	Cat#47-1172-82; RRID:AB_1582226; Clone ACK2
Anti-mouse CD11b (BV605)	BioLegend	Cat#101237; RRID:AB_11126744; Clone Mac-1
Anti-mouse CD11b (BUV395)	BD Biosciences	Cat#563553; RRID:AB_2738276; Clone M1/70
Anti-mouse CD11b (VioletFluor450)	Tonbo Biosciences	Cat# 75-0112; RRID:AB_2621936; Clone M1/70
Anti-mouse CD11c (APC)	BD Biosciences	Cat#550261; RRID:AB_398460; Clone HL3
Anti-mouse CD11c (BV605)	BD Biosciences	Cat#550261; RRID:AB_398460; Clone HL3
Anti-mouse CD11c (FITC)	BD Biosciences	Cat#553801; RRID:AB_395060; Clone HL3
Anti-mouse CD11c (PE)	Tonbo Biosciences	Cat#50-0114; RRID:AB_2621747; Clone N418
Anti-mouse CD135 (PE)	ThermoFisher	Cat#12-1351-82: RRID:AB_465859; Clone A2F10
Anti-mouse CD172a (BV605)	BD Biosciences	Cat#740390; RRID:AB_2740120; Clone P84
Anti-mouse CD19 (BV421)	BioLegend	Cat#115549; RRID:AB_2563066; Clone 6D5
Anti-mouse CD19 (BUV395)	BD Biosciences	Cat#563557: RRID:AB_2722495; Clone 1D3
Anti-mouse CD19 (PE-Cy7)	ThermoFisher	Cat#25-0193-82; RRID:AB_657663; Clone eBio1D3
Anti-mouse CD25 (APC)	ThermoFisher	Cat#17-0251-82; RRID:AB_469366; Clone PC61
Anti-mouse CD3e (BV421)	BioLegend	Cat#100336; RRID:AB_11203705; Clone 145-2C11
Anti-mouse CD4 (BV605)	BioLegend	Cat#100548; RRID:AB_2563054; Clone RM4-5
Anti-mouse CD4 (PE)	ThermoFisher	Cat#12-0042-82; RRID:AB_465510; Clone RM4-5
Anti-mouse CD44 (AF700)	BioLegend	Cat#103026; RRID:AB_493713; Clone IM7
Anti-mouse CD45 (BV570)	BioLegend	Cat#100548; RRID:AB_2562612; Clone 30-F11
Anti-mouse CD45.1 (APC-Cy7)	Tonbo Biosciences	Cat#25-0453; RRID:AB_2621629; Clone A20
Anti-mouse CD45.2 (APC)	ThermoFisher	Cat#17-0454-82; RRID:AB_469400; Clone 104
Anti-mouse Siglec-F (BV421)	BD Biosciences	Cat#562681; RRID: AB_2722581; Clone: E50-2440
Anti-mouse Ly-6C (BV711)	BioLegend	Cat#128037; RRID: AB_2562630; Clone: HK1.4
Anti-mouse CD45R (Violetfluor450)	Tonbo Biosciences	Cat#75-0452; RRID:AB_2621948; RA3-6B2
Anti-mouse CD49b (Pacific Blue)	BioLegend	Cat#108917; RRID:AB_2249376; Clone DX5
Anti-mouse CD62L (BV605)	BioLegend	Cat#104438; RRID:AB_2563058; Clone MEL-14
Anti-mouse CD90.2 (APC-eFluor780)	ThermoFisher	Cat#47-0902; RRID:AB_1272187; Clone 53-2.1
Anti-mouse CD90.2 (PE-Cy7)	BioLegend	Cat#140309; RRID:AB_10645336; Clone 53-2.1
Anti-mouse CX3CR1 (BV785)	BioLegend	Cat#149029; RRID:AB_2565938; Clone SA011F11
Anti-mouse Ly-6C (APC-eFluor780)	ThermoFisher	Cat#47-5932-82; RRID:AB_2573992; Clone HK1.4
Anti-mouse Ly-6G (PE-Cy7)	BioLegend	Cat#127618; RRID:AB_1877261; Clone 1A8
Anti-mouse MHC Class II (I-A/I-E) (redFluor710)	Tonbo Biosciences	Cat#80-5321; RRID:AB_2621997; Clone M5/114.15.2
Anti-mouse NK1.1 (eFluor 450)	ThermoFisher	Cat#48-5941-82; RRID:AB_2043877; Clone PK136
Anti-mouse Sca-1 (PE-Cy7)	BioLegend	Cat#25-5981-82; RRID:AB_469669; Clone D7
Anti-mouse Siglec-H (PE)	BioLegend	Cat#129606; RRID:AB_2189147; Clone 551
Anti-mouse TCR-β (APC-Cy7)	BioLegend	Cat#109220; RRID:AB_893624; Clone H57-597
Anti-mouse XCR1 (BV650)	BioLegend	Cat#148220; RRID:AB_2566410; Clone ZET
Anti-mouse XCR1 (PerCP-Cy5.5)	BioLegend	Cat#148208; RRID:AB_2564364; Clone ZET
Anti-mouse XCR1 (APC)	BioLegend	Cat#148206; RRID:AB_2563932; Clone ZET
Anti-mouse CD16/CD32	Tonbo Biosciences	Cat#70-0161; RRID: AB_2621487; Clone: 2.4G2
Anti-mouse TCR vβ5 (PE-Cy7)	BioLegend	Cat#139508; RRID:AB_2566021; Clone MR-94
Anti-mouse CD90.1 (FITC)	BD Biosciences	Cat#554894; RRID:AB_395585; Clone HIS51
Anti-mouse CD90.1 (APC)	ThermoFisher	Cat#17-0900-82; RRID:AB_469420; Clone HIS51
Anti-mouse Foxp3 (FITC)	ThermoFisher	Cat#11-5773-82; RRID:AB_465243; Clone FJK-16 s
Anti-mouse TCR-β (PerCP-Cy5.5)	BioLegend	Cat#109227; RRID:AB_1575176; Clone H57-597
Anti-mouse IL-17A (eFluor450)	ThermoFisher	Cat#48-7177-80; RRID:AB_11149677; Clone eBio17B7
Anti-mouse IFN-γ (FITC)	Tonbo Biosciences	Cat#35-7311; RRID:AB_2621724; Clone XMG1.2
Anti-mouse IL-4 (PE-Cy7)	TherrmoFisher	Cat#25-7042; RRID: AB_469674; Clone: BVD6-24G2
Anti-mouse CD3ε (PE-Cy7)	Tonbo Biosciences	Cat#60-0031; RRID:AB_2621824; Clone 145-2C11
Anti-mouse CD64 (PE-Cy7)	BioLegend	Cat#139314; RRID:AB_2563904; Clone X54-5/7.1
Anti-mouse CD64 (APC)	BioLegend	Cat#139306 RRID:AB_11219391; Clone X54-5/7.1
Anti-mouse CLEC12A (APC)	BioLegend	Cat#143406; RRID:AB_2564265; Clone 5D3/CLEC12A
Anti-mouse CD301 (PerCP-Cy5.5)	BioLegend	Cat#145710; RRID:AB_2564579; Clone LOM-14
Anti-mouse ESAM (APC)	BioLegend	Cat#136207; RRID:AB_2101658; Clone 1G8/ESAM
Anti-human AXL (AF488)	R&D Systems	Cat#FAB154G; RRID:AB_2714170; Clone 108724
Anti-human CD11c (PE-Cy7)	BioLegend	Cat#337216; RRID:AB_2129790; Clone BU15
Anti-human CD123 (BV711)	BioLegend	Cat#306030; RRID:AB_2566354; Clone 6H6
Anti-human CD14 (APC-Cy7)	BioLegend	Cat#325620; RRID:AB_830693; Clone HCD14
Anti-human CD14 (BV605)	BioLegend	Cat#301833; RRID:AB_11126983; Clone M5E2
Anti-human CD19 (BV605)	BioLegend	Cat#302244; RRID:AB_2562015; Clone HIB19
Anti-human CD19 (BV785)	BioLegend	Cat# 302239; RRID:AB_11218596; Clone HIB19
Anti-human CD19 (FITC)	BioLegend	Cat#302206; RRID:AB_314236; Clone HIB19
Anti-human CD1c (BV650)	BioLegend	Cat#331542; RRID:AB_2800866; Clone L161
Anti-human CD1c (PerCP-eFluor710)	eBioscience	Cat#46-0015-42; RRID:AB_10548936; Clone L161
Anti-human CD3ε (BV605)	BioLegend	Cat#317321; RRID:AB_11126166; Clone OKT3
Anti-human CD3ε (PE)	BioLegend	Cat#300308; RRID:AB_314044; Clone HIT3a
Anti-human CD45 (AF700)	BioLegend	Cat#304024; RRID:AB_493761; Clone HI30
Anti-human CD45 (PE-Cy7)	BioLegend	Cat# 304016; RRID:AB_314404; Clone HI30
Anti-human CD56 (BV605)	BioLegend	Cat#318334; RRID:AB_2561912; Clone HCD56
Anti-human CD56 (FITC)	BioLegend	Cat#318304; RRID:AB_604100; Clone HCD56
Anti-human CLEC10A (APC)	BioLegend	Cat#354706; RRID:AB_11219389; Clone H037G3
Anti-human CLEC4A	BioLegend	Cat#355306; RRID:AB_2561626; Clone 9E8
Anti-human FcεR1α (Percp)	BioLegend	Cat#334616; RRID:AB_2168079; Clone AER-37
Anti-human HLA-DR (APC-Cy7)	BioLegend	Cat#307618; RRID:AB_493586; Clone L243
Anti-human HLA-DR (BV605)	BioLegend	Cat#307640; RRID:AB_2561913; Clone L243
Anti-human XCR1 (BV421)	BioLegend	Cat#372610; RRID:AB_2687373; Clone S15046E
Lineage cocktail antibody	BD Biosciences	Cat#559971; RRID:AB_10053179
Anti-mouse Ly-6G eFluor450	eBioscience	Cat#48-5931-80; RRID:AB_1548797; Clone RB6-845
Anti-mouse TER-119 (eFluor450)	ThermoFisher	Cat#48-5921-82; RRID:AB_1518808; Clone TER-119

**Biological Samples**		

Melanoma	Memorial Sloan Kettering Cancer Center	See Table S6 for a list of patients included in this study.
Human HLA-DR+CD11c+ splenic cells, adult	Memorial Sloan Kettering Cancer Center	See Table S6 for a list of patients included in this study.

**Chemicals, Peptides, and Recombinant Proteins**		

Phorbol 12-myristate 13-acetate (PMA)	Millipore Sigma	Cat#P8139
Ionomycin calcium salt	Millipore Sigma	Cat#I0634
Brefeldin A	Millipore Sigma	Cat#B7651
Monensin sodium salt	Millipore Sigma	Cat#M5273
Ghost Dye Violet 510	Tonbo Biosciences	Cat#13-0870
Ghost Dye Red 780	Tonbo Biosciences	Cat#13-0865
Sytox Blue Dead Cell Stain	ThermoFisher	Cat#S34857
Trizol Reagent	ThermoFisher	Cat#15596018
CellTrace Violet Cell Proliferation Kit	ThermoFisher	Cat#C34557
OVA_323–339_ peptide	InvivoGen	Cat#vac-isq
Fixation/Permeabilization Solution Kit (Cytofix/Cytoperm)	BD Biosciences	Cat#554714
Transcription Factor Buffer Set	BD Biosciences	Cat#562574
123count eBeads	ThermoFisher	Cat#01-1234-42
Custom ProcartaPlex Multiplex Panel	ThermoFisher	Cat#PPX-18
AMPure XP beads	Beckman Coulter	Cat#A63881
Nextera XT DNA Library Prep kit	Illumina	Cat#FC-131-1024
Dynabeads Mouse DC Enrichment Kit	ThermoFisher	Cat#11429D
Dynabeads Human DC Enrichment Kit	ThermoFisher	Cat#11308D
CD4 T cell Isolation Kit, Mouse	Miltenyi Biotec	Cat#130-104-454
R848	Invivogen	Cat#tlrl-r848-5
S. enterica Lipopolysaccharide	Sigma-Aldrich	Cat#L6511
CpG (ODN 1826)	Invivogen	Cat#tlrl-1826-5
Recombinant murrine IFN-γ	PeproTech	Cat#315-05
Recombinant murine TNF-α	R&D Systems	Cat#410-MT-010
Recombinant murine IL-1β	PeproTech	Cat#211-11B
Recombinant murine IL-4	PeproTech	Cat#214-14
Recombinant murine IL-6	PeproTech	Cat#213-13-B
Recombinant murine IL-12	R&D Systems	Cat#419-ML-010
Collagenase A from Clostridium histolyticum	Sigma-Aldrich	Cat#11088793001
DNase I grade II, from bovine pancreas	Sigma-Aldrich	Cat#10104159001

**Critical Commercial Assays**		

Fixation/Permeabilization Solution Kit (Cytofix/Cytoperm)	BD Biosciences	Cat#554714
Transcription Factor Buffer Set	BD Biosciences	Cat#562574
123count eBeads	ThermoFisher	Cat#01-1234-42
Custom ProcartaPlex Multiplex Panel	ThermoFisher	Cat#PPX-18
AMPure XP beads	Beckman Coulter	Cat#A63881
Dynabeads Mouse DC Enrichment Kit	ThermoFisher	Cat#11429D
Dynabeads Human DC Enrichment Kit	ThermoFisher	Cat#11308D
CD4 T cell Isolation Kit, Mouse	Miltenyi Biotec	Cat#130-104-454
AMPure XP beads	Beckman Coulter	Cat#A63881
Nextera XT DNA Library Prep kit	Illumina	Cat#FC-131-1024
NEBNext® High-Fidelity 2X PCR Master Mix	New England Biolabs	Cat#M0541S
Chromium Single Cell 3′ Library & Gel Bead Kit V2	10X Genomics	Cat#120237

**Deposited Data**		

scRNA-seq	This paper	GEO: GSE137710
RNA-seq and ATAC-seq	This paper	GEO: GSE130201
Mouse genome assembly	UCSC Genome Browser	mm10.GRCm38
Gene annotations	GENCODE	vM17
Transcription factor motif database	CIS-BP	version 1.02
Immgen reference gene expression	Haemosphere	version 4.9.5
Gene ontology database	MSigDB	v7.0
Human mouse gene orthology	HGNC	HCOP
Human myeloid cells scRNA-seq	Villani et al., 2017	https://singlecell.broadinstitute.org/single_cell

**Experimental Models: Organisms/Strains**		

Mouse*: Tbx21*^*RFP-cre*^	Levine et al., 2017	N/A
Mouse*: Tbx21*^*RFP-creERT2*^	Levine et al., 2017	N/A
Mouse: *Rorc*^*cre*^ (B6.FVB-Tg(Rorc-cre)1Litt/J)	The Jackson Laboratory	Stock#022791
Mouse: *OTII (*B6.Cg-Tg(TcraTcrb)425Cbn/J)	The Jackson Laboratory	Stock#004194
Mouse: *Foxp3*^*Thy1.1*^	Liston et al., 2008	N/A
Mouse: *Ccr2*^–/–^	Provided by Frederic Geissmann (Sloan Kettering Institute, New York, USA)	N/A
Mouse*: R26*^*lsl-YFP-Ai3*^ (B6.Cg-*Gt(ROSA)26Sor*^*tm3(CAG-EYFP)Hze*^/J)	The Jackson Laboratory	Stock#007903
Mouse: C57BL/6J	The Jackson Laboratory	Stock#000664
Mouse: B6 CD45.1 (B6.SJL-Ptprc^a^ Pepc^b^/BoyJ)Mouse: C57BL/6J CD45.1	The Jackson Laboratory	Stock#002014
Mouse: *Ccr2*^*GFP*^	Provided by Eric Pamer (Sloan Kettering Institute, New York, USA)	N/A

**Software and Algorithms**		

FlowJo software	FlowJo, LLC	https://www.flowjo.com/
GraphPad Prism	Prism version 7	https://www.graphpad.com/scientific-software/prism; RRID: SCR_002798
HISAT2 v2.1.0	Johns Hopkins University	https://ccb.jhu.edu/software/hisat2/
R v3.4.0 (2017-04-21)	The Comprehensive R Archive Network	https://cran.r-project.org/
SAMtools v1.9	SourceForge	http://samtools.sourceforge.net/
Rsubread v1.28.1, v1.22.1	Bioconductor	https://bioconductor.org/packages/release/bioc/html/Rsubread.html
DESeq2 v1.22.1, v1.18.1	Bioconductor	https://bioconductor.org/packages/release/bioc/html/DESeq2.html
glmnet v2.0-16	The Comprehensive R Archive Network	https://cran.r-project.org/web/packages/glmnet/index.html
Bowtie2 v2.2.5	SourceForge	http://bowtie-bio.sourceforge.net/bowtie2/index.shtml
MACS2 v2.1.1.20160309	GitHub	https://github.com/taoliu/MACS
IDR v2.0.3	GitHub	https://github.com/nboley/idr
HOMER	UCSD	http://homer.ucsd.edu/homer/motif/
FIMO version 4.11.2	MEME suite	http://meme-suite.org/doc/download.html
SEQC	GitHub	https://github.com/ambrosejcarr/seqc
PhenoGraph	GitHub	https://github.com/jacoblevine/PhenoGraph
DoubletDetection	GitHub	https://github.com/JonathanShor/DoubletDetection
MAGIC	GitHub	https://github.com/dpeerlab/magic
f-scLVM	GitHub	https://github.com/bioFAM/slalom

### Lead Contact and Materials Availability

Further information and requests for reagents may be directed to, and will be fulfilled by, the Lead Contact: Alexander Rudensky (rudenska@mskcc.org). This study did not generate new unique reagents.

### Experimental Models and Subject Details

#### Mice

*Tbx21*^*RFP-cre*^ and *Tbx21*^*RFP-creERT2*^*, R26*^*lsl-YFP-Ai3*^*, Rorc*^*cre*^*, OTII, Foxp3*^*Thy1.1*^ mice have been previously described (Barnden et al., 1998, Eberl et al., 2004, Levine et al., 2017, Liston et al., 2008, Madisen et al., 2010). *Ccr2*^*GFP*^ mice were provided by E. Pamer. *Ccr2*^–/–^ mice were provided by F. Geissmann. C57BL/6 (CD45.1^+^) and C57BL/6 (CD45.2^+^) mice were purchased from Jackson Laboratories. *Tbx21*^*RFP-creERT2*^*R26*^*lsl-YFP*^ are homozygous for the *Tbx21* knock-in and heterozygous at the *R26*^*lsl-YFP*^ locus. *Tbx21*^*RFP-cre*^ mice are homozygous for the *Tbx21* knock-in. OTII-*Foxp3*^*Thy1.1*^ and *Rorc*^*cre*^*R26*^*lsl-YFP*^ mice are heterozygous at each locus. Generation and treatments of mice were performed under protocol 08-10-023 approved by the Sloan Kettering Institute (SKI) Institutional Animal Care and Use Committee. All mouse strains were maintained in the SKI animal facility in specific pathogen free (SPF) conditions in accordance with institutional guidelines and ethical regulations. For tamoxifen administration, 40mg tamoxifen dissolved in 100 μL ethanol and subsequently in 900 μL olive oil (Sigma-Aldrich) were sonicated 4 × 30 s in a Bioruptor Twin (Diagenode). Mice were orally gavaged with 200 μL tamoxifen emulsion per treatment. For antibiotic treatment, mice were weaned onto filtered antibiotic-treated water containing ampicillin, kanamycin, vancomycin (0.1% w/v each) and metronidazole (0.05% w/v).

Both male and female mice were included in the study and we did not observe sex-dependent effects. All mice analyzed were sex and age matched (6–10 weeks old). All animals used in this study had no previous history of experimentation and were naive at the time of analysis.

#### Human specimens

Human tissue samples (Table S6) were obtained in accordance with national guidelines. All patients signed informed consent and the study was approved by the institutional review board (IRB) at Memorial Sloan Kettering Cancer Center. Tumor tissue was collected from surgical specimens after macroscopical examination of the tissue by a pathologist. For each specimen, a fragment was formalin-fixed and paraffin embedded (FFPE) for histology. The remainder of the tissue was directly processed to obtain single cell suspensions.

Spleen tissues were obtained from patients undergoing resection of tumors in other organs. Patients were not subjected to chemotherapy or immunotherapy prior to surgery and the spleens were macroscopically normal.

Human PBMCs were isolated from Buffy coats from healthy donors and cryopreserved.

### Method Details

#### Cell isolation

Mice were euthanized by CO_2_ inhalation. For analysis of DCs from lung or liver, mice were immediately perfused with 20 mL ice-cold PBS. Organs were harvested and processed as follows. Lymphoid organs, lung and liver were digested in collagenase in RPMI1640 supplemented with 5% fetal calf serum, 1% L-glutamine, 1% penicillin–streptomycin, 10 mM HEPES, 1 mg/ml collagenase A (Sigma, 11088793001) and 1U/mL DNase I (Sigma, 10104159001) for 45 min at 37°C, 250 rpm. Small and large intestine were removed, flushed with PBS and after Peyer’s patches were removed 0.5-cm-long fragments of intestines were further washed in PBS and incubated in PBS supplemented with 5% fetal calf serum, 1% L-glutamine, 1% penicillin–streptomycin, 10 mM HEPES, 1 mM dithiothreitol, and 1 mM EDTA for 15 min to remove the epithelial layer. Samples were washed and incubated in digest solution for 30 min. 1/4 inch ceramic beads (MP Biomedicals, 116540034) were added to large intestine samples (3 per sample) to aid in tissue dissociation. Digested samples were filtered through 100-μm strainers and centrifuged to remove collagenase solution. Cells from lung, liver and intestinal lamina propria were resuspended in 40% Percoll and centrifuged to remove debris. For BM isolation, the femur and tibia, after the tips of the bones were removed, were placed in a punctured PCR tube positioned within a 1.5ml Eppendorf tube. Samples were pulse spun to pellet BM cells at the bottom of the Eppendorf tube, washed in PBS-2% FBS, filtered through a 100-μm strainer and kept on ice until ready for further use. For *in vitro* co-culture experiments, splenic CD4^+^ T cells were enriched using the Miltenyi CD4 Negative Selection Isolation Kit (Miltenyi). Single cell suspensions of human PBMCs or splenocytes were thawed prior to flow cytometric analysis.

#### Preparation of human tissue cell suspensions

Spleen tissue was dissociated by manual mincing followed by an incubation of 45 min at 37°C, 250 rpm, in RPMI1640 supplemented with 5% fetal calf serum, 1% L-glutamine, 1% penicillin–streptomycin, 10 mM HEPES, 1 mg/ml collagenase A (Sigma, 11088793001) and 1U/mL DNase I (Sigma, 10104159001). Cells were then washed and filtered with a 100 μm filter. Cells were either immediately processed for DC enrichment and cell-sorting (scRNA-seq) or cryopreserved in Bambanker media and cryopreserved.

Fresh tumor tissue was dissociated by manual mincing followed by an incubation of 30 min at 37°C in RPMI with liberase (0.83mg/ml; Sigma-Aldrich), alternated with 3 rounds of dissociation with a gentleMACS™ dissociator (Miltenyi). After dissociation, cell suspensions were filtered with a 100 μm filter, and resuspended in ACK lysis buffer before washing with RPMI-5% FBS. Cells were then washed with PBS and stained with LIVE/DEAD Fixable Ghost Dye Red 780 followed by anti-CD45. Viable immune cells were sorted on a FACS Aria II sorter (BD Biosciences).

#### Flow cytometry analysis

To assess cytokine production after *ex vivo* restimulation, single cell suspensions were incubated for 3 hours at 37°C with 5% CO2 in the presence of 50ng/mL PMA and 500 ng/mL ionomycin with 1 μg/mL brefeldin A and 2 μM monensin. For flow cytometric analysis, dead cells were excluded either by staining with SYTOX blue (Invitrogen) after cell-surface staining (live cells) or cells were washed with cold PBS and then stained with LIVE/DEAD Fixable Violet or Ghost Dye Red 780 in PBS for 10 minutes at 4°C, prior to cell-surface staining. Cells were then incubated with anti-CD16/32 in staining buffer (2% FBS, 0.1% Na azide, in PBS) for 10 minutes at 4°C to block binding to Fc receptors. Extracellular antigens were stained for 20-30 minutes at 4°C in staining buffer. Cells were fixed and permeabilized with BD Cytofix/Cytoperm (for cytokine analysis) or BD Transcription Factor Fix/Perm (for transcription factor analysis) per manufacturer instructions. Intracellular antigens were stained for 30min at 4°C in the appropriate 1x Perm/Wash buffer. Cells were washed with staining buffer before acquisition on a BD LSR II flow cytometer (Becton Dickinson) or Cytek Aurora . 123count eBeads were added to quantify absolute cell numbers.

#### Bone Marrow Progenitor Analysis

For sorting, BM cell suspensions were depleted of Lin^+^ cells with a cocktail of biotin-conjugated antibodies [anti-CD3, B220 and NK1.1 for MDP and CDPs or anti-CD3, B220, NK1.1, Gr-1 and CD11b for pre-DCs; Biotin Lineage Mouse Panel (BD Biosciences)] and anti-biotin microbeads (Miltenyi) followed by separation on an LS column (Miltenyi). After staining for analysis or cell sorting, MDPs were identified as Lin(B220, CD3, NK1.1, Ter-119, Gr1, CD11b)^–^Sca1^–^MHCII^–^CD11c^–^CD135^+^CD115^+^CD117^hi^; CDPs were identified as Lin(B220, CD3, NK1.1, Ter-119, Gr1, CD11b)^–^Sca1^–^MHCII^–^CD11c^–^CD135^+^CD115^+^CD117^int/lo^; pre-DCs were gated as Lin(B220, CD3, NK1.1, Ter-119)^–^Sca1^–^CD11c^+^MHCII^–^CD135^+^CD172α^–^. 1-2 × 10^4^ FACS-isolated DC precursors were transferred via intravenous retro-orbital injection into three-week old CD45.1 recipients. Spleens were harvested 7 days after transfer and the phenotype of the CD45.2^+^ progeny of the transferred cell populations was analyzed by flow cytometry.

#### Adoptive transfer of splenic Siglec H^+^ pre-DCs

*Tbx21*^*RFP-cre*^CD45.2^+^Ly6C^−^CD64^–^MHCII^+^CD11c^+^Siglec-H^+^ cells were transferred via intravenous retro-orbital injection into six-week old sublethally irradiated (650 rad) CD45.1 recipients. At 7 d after cell transfer, spleens were collected and single-cell suspensions were analyzed by flow cytometry to establish the phenotype of the CD45.2^+^ progeny.

#### *In vivo* LPS immunization

Mice were immunized with 2 μg of LPS, intra-peritoneally. After 16 hr, spleens were harvested and single-cell suspensions analyzed by flow cytometry.

#### *In vitro* DC stimulation

5 × 10^4^ sort-purified DCs were cultured in 96-well plates in the presence of R848 (Invivogen 2.5 μg/ml), LPS (100ng/ml; Sigma-Aldrich), CpG (1 μm; ODN 1826 Invivogen), IFN-γ (20ng/ml; Peprotech) or TNF-α (20ng/ml; R&D Systems) for 16 hours.

#### DC Cytokine production

5 × 10^4^ sort-purified DCs were cultured in 96-well plates in the presence of CpG (5 μm; ODN 1826 Invivogen), or R848 (2.5 μg/ml; Invivogen) for 18 hours, triplicate wells per condition. Cell-free supernatants were then harvested and stored at −80°C. These were thawed immediately prior to analysis using a custom multiplexed ProcartaPlex assay, carried out according to manufacturer instructions with technical duplicates and read on a Luminex 200 (Luminex Corp) instrument. Standard curves were generated and values interpolated using Graphpad Prism.

#### Cytospin analysis

Cytospins of 3 × 10^4^ – 5 × 10^4^ sort-purified cells were stained with May–Gruenwald solution (VWR), washed and stained with Giemsa (Fisher Scientific) for 20min. Slides were air-dried and sealed with mounting medium, and images were taken with a 40X Mirax scanner.

#### *In vitro* cell culture

Naive TCRβ^+^CD4^+^vβ5^+^Thy1.1(Foxp3)^–^CD44^lo^CD62L^hi^ OTII T cells were sort purified after enrichment with a CD4^+^ T cell negative selection kit (Miltenyi Biotec). DC subsets were sort purified after enrichment with Dynabeads Mouse DC Enrichment Kit (Invitrogen). DCs and T cells were co-cultured in triplicate at a ratio of 2 × 10^4^ DCs to 1 × 10^5^ T cells in the presence of OVA_323–339_ (1 μg/ml; Invivogen), under Th0 conditions (anti-IL-4 (11B11) and anti-IFN-γ (XMG1.2)) or with the addition of polarizing cytokines (Th1: 10 ng/ml IL-12 (R&D systems) and anti-IL-4; Th2: 10 ng/ml IL-4 (Peprotech), anti-IL-12 (C17.8), and anti-IFN-γ; Th17: 2ng/ml TGF-β1 (R&D systems), 20 ng/ml IL-6 (Peprotech), 10 ng/ml IL-1β, anti-IL-4, and anti-IFN-γ; iTreg: 2ng/ml TGF-β1, 500IU/ml of IL-2 (Peprotech). IL-2 (50IU/ml) was added to Th0, Th1, and iTreg conditions on d3. All blocking antibodies were at 10 μg/ml. Cytokine production or Foxp3 expression was assessed after 4 days of culture.

#### *In vitro* T cell proliferation assays

1 × 10^5^ labeled sort-purified naive TCRβ^+^CD4^+^Thy1.1(Foxp3)^–^CD44^lo^CD62L^hi^ OTII T cells were co-cultured with 2 × 10^4^ sort-purified DCs in the presence of OVA_323–339_ (1 μg/ml), or left unstimulated. OT-II T cells were labeled with CellTrace Violet (Thermo Fisher Scientific) according to the manufacturer’s instructions. Dilution of the cell dye was assessed by flow cytometry on day 5 of culture.

#### Single-cell RNA sequencing

Mouse DC subsets were enriched from a pool of two spleens with Dynabeads Mouse DC Enrichment Kit (ThermoFisher). Lin(CD3, CD19, CD90)^–^CD64^–^Ly6C^–^CD11c^+^MHCII^+^ cells were then purified using an Aria II cell sorter (BD Bioscience) into two populations (RFP^+^ and RFP^–^ cells). Cells were sorted into cRPMI, before being pelleted and resuspended in RPMI-2% FBS. Human DCs from spleen were enriched with Dynabeads Human DC Enrichment kit (ThermoFisher). Lin(CD3, CD19, CD56)^–^CD14^–^CD11c^+^MHCII^+^ cells were then FACS-isolated into RPMI-2% FBS for single cell RNA-seq. Human CD45^+^ cells from human melanoma samples were FACS-isolated into RPMI-2% FBS for single cell RNA-seq. The scRNA-seq libraries were prepared following the user guide manual (CG00052 Rev E) provided by the 10X Genomics and Chromium Single Cell 3′ Reagent Kit (v2). Briefly, samples were encapsulated in microfluidic droplets at a dilution of ∼70 cells/μl (doublet rate ∼3.9%.). Encapsulated cells were subjected to reverse transcription (RT) reaction at 53°C for 60 min. After RT step, the emulsion droplets were broken and barcoded-cDNA was purified with DynaBeads, followed by 14-cycles of PCR-amplification (98°C for 180 s; [98°C for 15 s, 67°C for 20 s, 72°C for 60 s] x 12-cycles; 72°C for 60 s). 50 ng of PCR-amplified barcoded-cDNA was fragmented with the reagents provided in the kit and purified with SPRI beads to obtain an average fragment size of 600 bp. Next, the DNA library was ligated to the sequencing adaptor followed by indexing PCR (98°C for 45 s; [98°C for 20 s, 54°C for 30 s, 72°C for 20 s] x 10 cycles; 72°C for 60 s). The resulting DNA library was double-size purified (0.6-0.8X) with SPRI beads and sequenced on an Illumina NovaSeq platform (R1 – 26 cycles, i7 – 8 cycles, R2 – 96 cycles) resulting in 184.5-186.1 million reads per sample (average reads per single-cell being 42,000 and average reads per transcript 4.40-7.14).

#### Bulk RNA-sequencing

Bulk transcriptomes were generated from cells pooled from two mice with two (MLN) or three (spleen) biological replicates. Lin(CD3, CD19, CD90)^–^CD64^–^Ly6C^–^CD11c^+^MHCII^+^ cells were sorted into the following three populations: CD11b^+^XCR1^−^RFP^−^ (T-bet^+^ cDC2), CD11b^+^XCR1^−^RFP^+^ (T-bet^–^ cDC2) and CD11b^−^XCR1^+^ (cDC1), and resuspended in Trizol. RNA was extracted and after 12 cycles of amplification using the SMART-Seq v4 Ultra Low Input RNA Kit (Clonetech catalog # 63488) libraries were prepared using the KAPA Hyper Prep Kit (Kapa Biosystems KK8504) by the Integrated Genomics Core (MSKCC). Samples were barcoded and run on a HiSeq 4000 or HiSeq 2500 in rapid mode in a 50bp/50bp paired end run, using the HiSeq 3000/4000 SBS Kit or HiSeq Rapid SBS Kit v2 (Illumina). An average of 30 million paired reads were generated per sample and the percent of mRNA bases per sample ranged from 70 - 80%.

#### ATAC-sequencing

5 × 10^4^ sort-purified DCs were collected and ATAC-seq libraries were prepared as previously reported (Buenrostro et al., 2015) with the following modifications. Cells were washed with 50 μL of PBS and cell pellets were resuspended in 50 μL of cold lysis buffer followed by centrifugation. Cell pellets were resuspended in 50 μL of Tn5 transposase mixture: 1x Tagment DNA Buffer, 2.5 μL Tagment DNA Enzyme (Nextera DNA Library Preparation Kit, Illumina). Cells were incubated at 42°C for 45 minutes with agitation followed by DNA isolation using the MinElute Reaction Cleanup Kit (QIAGEN, Hilden, Germany). Construction of ATAC-seq libraries included an initial round of PCR in a total volume of 50uL using the NEBNext High-Fidelity 2X PCR Master Mix (New England Biolabs, MA, USA) with primers (1.25 μM each) from Buenrostro et al. (2015) with the following thermal cycles: 5 minutes at 72°C, 30 s at 98°C, followed by 5 cycles [98°C for 10 s, 63°C for 30 s and 72°C for 60 s]. To avoid over amplification of libraries which result in GC bias, 5uL of the PCR-amplified DNA were subjected to qPCR (BioRad Real-Time PCR System) in a volume of 15uL using SYBR Green dye (final 0.6x SYBR Green I, Life Technologies) and with the respective primers (1.25 μM each). Following qPCR [30 s at 98°C, followed by 30 cycles (98°C for 10 s, 63°C for 30 s and 72°C for 60 s)], amplification curves were analyzed and the optimal number of PCR cycles for each sample were estimated with cycle thresholds reaching 1/3 of the maximum. Upon selecting the cycle threshold, the remaining 45uL of the initial PCR mix was subjected to a second round of PCR with the following thermal cycles: 30 s at 98°C, followed by n cycles [98°C for 10 s, 63°C for 30 s and 72°C for 60 s]. The libraries were purified by Agencourt AMPure XP beads (x1.8 vol.). Samples were quantified and prepared for sequencing by the Integrated Genomics Core at Memorial Sloan Kettering Cancer Center. After PicoGreen quantification and quality control by Agilent BioAnalyzer, libraries were pooled equimolar and run on a HiSeq 4000 in a 50bp/50bp paired end run, using the HiSeq 3000/4000 SBS Kit (Illumina). The average number of read pairs per sample was 122 million

### Quantification and Statistical Analysis

#### Mouse scRNA-seq Data Analysis

Data from scRNA-seq of RFP^–^ and RFP^+^ samples were individually processed using the SEQC pipeline (Azizi et al., 2018) using the package provided mm38 mouse genome reference and default parameters for the 10X platform. The SEQC pipeline performs read alignment, multi-mapping read resolution, as well as cell barcode and UMI correction to generate a (cells x genes) count matrix. The pipeline further performs the following initial cell filtering steps: true cells are distinguished from empty droplets based on the cumulative distribution of total molecule counts; cells with a high fraction of mitochondrial molecules are filtered (> 20%); and cells with low library complexity are filtered (cells that express very few unique genes). The resulting count matrix from SEQC contained 5931 cells by 15084 genes, after 4.13% RFP^+^ cells and 1.44% RFP^–^ cells were filtered for high mitochondrial content and 0.39% RFP^+^ cells and 1.8% RFP^–^ cells were filtered for low library complexity. These default filters were designed to be permissive and cells that express < 1000 unique genes were further filtered to remove any remaining low quality cell libraries (determined by inspection of the bimodal log10 genes expressed count distribution). Genes that were expressed in more than 10 cells were retained for further analysis. The resulting filtered count matrix (4464 cells by 11755 genes) contained 2032 RFP^+^ cells with a median of 6146 molecules/cell and 2432 RFP^–^ cells with a median of 10263 molecules/cell.

This filtered count matrix was normalized for library size. This normalized matrix was then multiplied by the median of the total molecule count across all cells for numerical stability and log2 transformed with a pseudocount of 0.1 for downstream analysis. Principal Component Analysis (PCA) was applied to the data, selecting the 20 top PCs based on the decay in explained variance per additional PC. While the point of maximum curvature suggested a threshold of 8 PCs, we conservatively included 20 PCs (explaining 13% of the total variance) and retained the reduced matrix for further downstream analysis.

For clustering analysis, to ensure capture of smaller discrete populations and a fine clustering across our dataset we applied Phenograph to the PCA reduced expression matrix with k = 40. To evaluate the clustering robustness to input parameters of the discrete subpopulations, we separately evaluated robustness in two partitions of the data: a partition containing cells in the discrete subpopulations (clusters 3, 10, 11, 12, 14, 16) and a partition containing cells in the more continuous cDC1 and T-bet^+^ cDC2 populations (Figure S7A). For the discrete subpopulations, as we swept input parameters, in each clustering solution we consolidated clusters with majority membership in either cDC1 or T-bet^+^ cDC2 populations, and then calculated the adjusted Rand Index (RI) between pairs of these solutions (Figure S7A). For the input number of PCs ranging from 10 to 60 and k ranging from 10 to 60, the partitioning between these discrete subpopulations was robust (RI_PC_ = 0.95 ± 0.029, RI_k_ = 0.95 ± 0.022). For the complementary partition, where we consolidated clusters with majority membership in the discrete subpopulations, the clustering solutions were less robust (RI_PC_ = 0.75 ± 0.13, RI_k_ = 0.73 ± 0.087; Figure S7A), indicating that heterogeneity among cDC1s and T-bet^+^ cDC2s was not coherently described by clustering. At higher numbers of PCs and higher k adjacent T-bet^+^ and Tbet^–^ cDC2 clusters tended to collapse, likely reflecting a considerable degree of continuity between these phenotypes as we describe using Palantir and diffusion maps (below).

For Phenograph cluster annotation, we used bulk gene expression profiles of sorted myeloid cells reported by the Immgen Consortium (Miller et al., 2012). Given that our sorting strategy is selective for CD11c^+^ splenic DCs, we restricted our comparison to splenic DCs and related populations in the Immgen database (Figure S1E). Bulk samples were library-size normalized and z-scored. We computed the mean gene expression for each of the Phenograph clusters and standardized expression values (z-score). Cluster centroids were then correlated with bulk profiles. Pearson correlation coefficients are shown for each pairwise comparison between datasets (Figure S1C). We found that clusters 1, 4, 5, 7, 9, and 13 are all maximally correlated with ImmGen CD8^+^ splenic DCs and are accordingly labeled cDC1, supported by high expression of *Xcr1*, *Clec9a*, and *Irf8*. Similarly, T-bet^+^ clusters (0, 2, 6, 8, and 15) and cluster 14 (a mix of T-bet^+^ and T-bet^–^ DCs) are maximally correlated with ImmGen CD4^+^ splenic DCs and are labeled cDC2, supported by high expression of *Sirpa*, *Cd4*, and *Irf4*. Cluster 12 correlated highly with Immgen skin-draining lymph node DCs corroborating their annotation as a migratory DC subset. Cluster 16 showed a high correlation with ImmGen monocyte profiles. Further examination of post-sort purity and expression of marker genes indicated that this is contaminating population of CD11c^–^MHCII^–^Ly6C^+^ monocytes. While clusters 3 and 10 correlated most highly with ImmGen macrophage and monocyte profiles, their expression of *Itgam* (CD11b), *Sirpa* and *Zbtb46* corroborates their cDC2 annotation. Doublet detection (http://gitub.com/JonathanShor/DoubletDetection) indicated that the cDC1 cluster 9 may consist of cDC1/cDC2 doublets. We retained this cluster in our characterization as we cannot rule out the possibility they may be phagocytic cells. To characterize proliferative phenotypes across the clusters, for each cell we calculated the average expression of established gene expression signatures for G1/S and G2/M cell-cycle phases (Tirosh et al., 2016). The distribution of the single-cell expression of these signatures were markedly increased in T-bet^+^ clusters 6 and 8 as well as in cDC1 cluster 4 (Figure 1H).

To account for some missing values in scRNA-seq, we employed MAGIC, a method of “de-noising” and imputing missing expression values through data diffusion between cells with similar covariate gene relationships (van Dijk et al., 2018). We constructed the adaptive affinity matrix using k = 30, ka = 10, and t = 4 as input parameters, where t specifies the number of times the affinity matrix is powered for diffusion. As previously demonstrated (van Dijk et al., 2018), MAGIC imputed values are robust to specific choice of these input parameters. We found for k ranging from 20 to 60 and for t ranging from 3 to 12, the median R^2^ across all genes was at least 0.90 between imputed expression solutions (Figure S7B). Following MAGIC, gene expression values were no longer sparse and followed better structured distributions that align to the data manifold. These distributions can then be directly used to assess expression differences between cell subsets (van Dijk et al., 2018), thus taking advantage of the entire distribution of values in the comparison.

To quantify the difference between distributions, we use the earth mover’s distance (EMD, also known as the Wasserstein distance). EMD, which quantifies the amount of work required to transport one distribution to another and in one dimension, was calculated as the L1 norm of the cumulative distribution functions under comparison, EMD = ||CDF1 - CDF2||1. We assigned a positive or negative EMD score according to directionality of the change in the medians of the two distributions. To identify distinguishing marker genes for a given cluster, we computed the EMD score for each gene, comparing the imputed expression within the cluster to the imputed expression in all cells outside the cluster (one-versus-rest). We additionally directly compared the expression distributions of T-bet^+^ cDC2 to T-bet^–^ cDC2. Given that cell numbers were similar to an order of magnitude in our clusters, rescaling EMD measures to account for differences in cell numbers between clusters had little effect on our results.

To further identify discriminative markers for each cluster, we also evaluated the classification performance of individual genes in one-versus-rest comparisons. Specifically, for a given cluster, we evaluated how well the imputed expression of a gene can discriminate between cells within the cluster from all cells outside the cluster using the area under the ROC curve (AUC) score. The AUC score summarizes the performance of a gene threshold based classifier. Genes with an AUC score near 1 are strong positive discriminators (expression positively associated with the cluster), while genes with an AUC score near 0 are strong negative discriminators (expression negatively associated with the cluster). We used imputed expression for the calculation of AUC scores to retain the discriminative potential of genes with sparse expression. We reasoned that a combination of EMD and AUC score indicate strong discriminative markers for identified clusters. A large EMD score for a gene indicates large expression differences between clusters, while an AUC score near 0 or 1 indicates gene expression that strictly discriminates the clusters.

For cell cycle-related gene expression correction, we applied f-scLVM (Buettner et al., 2017) to factor out their effects from our expression values. f-scLVM relies on provided input ontologies to seed a latent factor model for the observed expression matrix. We used the following gene ontologies retrieved from MSigDB (Subramanian et al., 2005) to annotate the cell cycle effect: GO:0000279 M phase, GO:0006260 DNA replication, GO:0007059 chromosome segregation, GO:0000087 M phase of mitotic cell cycle, GO:0048285 organelle fission. To provide starting points for f-scLVM to model other relevant latent factors in our dataset along with cell-cycle effects, we additionally provided a set of ontologies derived from principal components. For each of our first 20 principal components, we created a gene set to input as a factor to f-scLVM by concatenating the top 30 positive and negative gene loadings (60 total) associated with that PC. We found this was necessary to prevent f-scLVM from adjusting cell-cycle factors to capture other signals in the data. We fitted the f-scLVM model on the normalized and log expression values for cDC2 clusters with otherwise default input parameters. In the resulting model, we considered each input factor with primarily cell-cycle annotated genes among its top terms as a cell-cycle effect. We then regressed these cell-cycle effects from the expression of each gene individually in the cDC2 expression matrix through multiple linear regression using default functions provided by f-scLVM.

#### Diffusion maps and Palantir analyses

We considered T-bet^+^ cDC2 clusters, T-bet^–^ cDC2 clusters, and the Siglec-H^+^ DC cluster for trajectory analysis focused on the transition from T-bet^–^ cDC2 to T-bet^+^ cDC2. We generated diffusion maps following the strategy outlined in Setty et al. (2018). From the selected clusters, we used cell-cycle corrected expression values to construct an adaptive affinity matrix as described for MAGIC imputation above. Eigenvectors of this affinity matrix are termed diffusion components. Based on the eigen gap, we chose to use 7 diffusion components for downstream use in Palantir and for calculating diffusion distances. We scale each included diffusion component by the factor λ/(1-λ) where λ is the associated eigenvalue, to reflect ‘multi-scale’ diffusion distances.

We used Palantir to characterize potential pseudo-time trajectories emanating from cells belonging to the Siglec-H^+^ cluster. Given a starting input cell, Palantir models cell fate as a continuous probabilistic process and calculates each cell’s position in pseudotime along with the probability that each cell reaches each terminal state, termed the branch probability. Palantir also reports the differentiation potential of each cell, measured by the entropy of branch probabilities associated with the cell. Cells in the Siglec-H^+^ cluster were distinguished by Diffusion Component 3. We chose the cell with the most extreme value as the input start cell. We ran Palantir using the 7 diffusion components described above, k = 20 and 2000 waypoints to sufficiently cover all clusters for pseudotime calculation. All 5 terminal points shown in Figure S5B were automatically identified by Palantir and are corroborated by diffusion component extrema. Reported gene expression trends in pseudotime were calculated using default methods provided by Palantir, which fits a generalized additive model weighted by branch probabilities to MAGIC imputed expression values.

To further illustrate the relationships between Siglec-H^+^ DC, T-bet^+^ and T-bet^–^ cDC2 clusters, we projected cells from each set of clusters onto their corresponding top two diffusion components (Figure S4D). These projections demonstrate that phenotypic extrema from these clusters are spanned by a continuum of states. The projection of Siglec-H^+^ DC and T-bet^+^ cluster cells on primary diffusion components (Figure S4D, left panel) additionally illustrates connectivity between Siglec-H^+^ DC cells with proliferative cells from the T-bet^+^ clusters 6 and 8, further supporting the potential progenitor capabilities of the Siglec-H^+^ DC. Additionally, we calculated shortest paths from the Siglec-H^+^ DC starting cell to all terminal cell states used in Palantir above based on k-NN graphs of diffusion distances (k = 15). Shortest path step sizes from the Siglec-H^+^ DC to all terminal states do not contain apparent outliers, indicating continuity between the states (Figure S5F, bottom panel). We evaluated cells in proximity to shortest paths by randomly sampling shortest paths from k-l-NN graps, where l edges are randomly subsampled for each cell in the k-NN graph (k = 15, l = 5). We calculated the probability that each cell is within 20 nearest neighbors of the sampled paths (Figure S4F, top panel), as well as the proportion of cells along the path belonging to the Siglec-H^+^ DC, T-bet^+^ cDC2 or T-bet^–^ cDC2 subset (Figure S5F, middle panel). Shortest paths from the Siglec-H^+^ DC toward T-bet^+^ states do not pass through T-bet- clusters and vice versa (Figure S4F, middle panel), indicating that both cDC2 subsets may arise directly from a common Siglec-H^+^ DC progenitor in the spleen.

#### Human scRNA-seq

Human splenic DCs were processed using the 10X Genomics Chromium System (Chromium Single Cell 3′ Reagent Kits User Guide v2 Chemistry). FASTQ files were processed using the Sequence Quality Control (SEQC) pipeline (Azizi et al., 2018), as described above, and reads were aligned to the human genome hg38. A total of 0.72% and 0.02% of cells were filtered out due to high mitochondrial RNA fraction and low molecule complexity respectively, resulting in 5110 cells/sample and a median of 9656 molecules/cell. Raw count libraries were additionally filtered to retain cells with greater than 6000 total molecules to remove low quality libraries (determined by bimodal library size count distribution), removing an additional 7.6% of cells. Genes detected in greater than 10 cells were retained for further analysis. The filtered count data (4721 cells by 12476 genes) was normalized for library size and log2 transformed. Phenograph was used to cluster PCA reduced expression data, using k = 30 and 20PCs, explaining 6.8% of the data variance. Application of Doubledetection (v2.4 https://zenodo.org/record/2678042) identified two clusters that contained a majority of putative cDC1 and cDC2 doublets that were subsequently removed. A small cluster (31 cells) of putative ILC3 contaminants were also removed, resulting in a remaining total of 4465 cDC cells. Differential expression analysis was performed using EMD, as defined above, calculated on normalized, log transformed and MAGIC imputed data (van Dijk et al., 2018). Diffusion maps were calculated for the cDC2 subset as described above.

Single cell transcriptomes from tumor associated human cDC2s were analyzed from FACS-isolated CD45^+^ cells from human melanoma samples (n = 2), either fresh or after short term storage at −80C in Bambanker (Lymphotec, Inc.). Samples were processed using the 10X Genomics Chromium System (Chromium Single Cell 3′ Reagent Kits User Guide v2 Chemistry). FASTQ files were processed using the Sequence Quality Control (SEQC) pipeline (Azizi et al., 2018), as described above, and reads were aligned to the human genome hg38. A median of 12.14% and 2.9% of cells were filtered out due to high mitochondrial RNA fraction and low molecule complexity respectively, resulting in a median of 3856 cells/sample and 2827 molecules/cell. Raw count libraries were additionally filtered to retain cells with greater than 1000 total molecules to remove low quality libraries (determined by bimodal library size count distribution), removing an additional 11.4% of cells. Genes detected in greater than 10 cells were retained for further analysis. The filtered count data (9315 cells by 15040 genes) was normalized for library size and log2 transformed. Phenograph was used to cluster PCA reduced expression data, using k = 30 and 20PCs, explaining 14.8% and 18.5% of data variance in complete cohort and in myeloid subsets, respectively. For cluster annotation, Pearson correlation coefficient was calculated as described above to assess similarity of Phenograph clusters centroids and bulk RNA-seq subsets (Jeffrey et al., 2006, Novershtern et al., 2011). Residual melanoma cells after CD45^+^ sorting were identified by tyrosinase (TYR) positivity. Differential expression analysis was performed using EMD, as defined above, calculated on normalized, log transformed and MAGIC imputed data (van Dijk et al., 2018). MAGIC imputed expression of canonical myeloid genes and analysis of differentially expressed genes were used to determine cell identity of myeloid cells (n = 2122 cells).

#### Analysis of human blood myeloid single cell transcriptomes

We utilized publicly available single-cell transcriptomic data from FACS-isolated human peripheral blood DCs and monocytes, generated with a Smart-Seq2 based protocol (Villani et al., 2017). Normalized and log transformed data were downloaded from the Broad Single-cell portal along with corresponding cluster annotations.

#### Correspondence between mouse and human DC scRNA-seq subsets

To characterize whole transcriptome correspondence between mouse and human cDC2 subsets, we computed cluster centroids in both mouse and human datasets for orthologous genes identified using the HGNC database (Seal et al., 2011). We then standardized (z-score) expression values for each gene across the cluster average profile within each dataset before calculating the Pearson correlation coefficient for each pairwise mouse-human cluster comparison.

To analyze the correspondence of transcription regulator expression between mouse and human cDC2 subsets, we independently clustered transcriptional regulator expression patterns and examined their overlap across species. We collected a comprehensive transcription factor annotation from AnimalTFDB 3.0 (Hu et al., 2019), retaining 662 orthologous TFs that passed our predefined gene expression threshold in both species. We selected top varying transcriptional regulators by one-way ANOVA on library-size normalized log-transformed expression across cDC2 clusters. After Benjamini-Hochberg p value correction, 103 transcription factors passed an FDR threshold of 1e-5 in both species. The MAGIC imputed expression profiles of these transcription factors were z-scored and clustered in each species separately using Phenograph with k = 10 and the cosine distance metric. Transcription factor gene clusters were associated with cDC2 subsets in each species by their maximal collective expression across cDC2s.

#### Bulk RNA sequencing analysis

Mouse genome assembly mm10.GRCm38 was used for bulk RNA-seq read alignments. Gene annotations from GENCODE vM17 were used for all analysis. Analysis was done using custom scripts in R v3.4.0 (2017-04-21). Reads were aligned to the mouse genome assembly mm10.GRCm38 using HISAT2 v2.1.0 (Kim et al., 2015), with default parameters including splice sites obtained from gene annotations. Uniquely aligned reads were extracted using grep with parameters “-v 'NH:i:[2-9]'” and SAMtools v1.9 (Li et al., 2009), with parameters “view -h -F 4 -q 20 -b” and sorted and indexed using SAMtools. For analysis of RNA-seq samples in cDC1s, T-bet^–^ cDC2s, T-bet^+^ cDC2s in the spleen and the lymph node, reads aligned to genes from the GENCODE gene annotation were counted using Rsubread v1.28.1 (Liao et al., 2013). Differential gene expression between any pair of samples was assessed using DESeq2 v1.22.1 (Love et al., 2014), using default FDR adjustment of p values for multiple hypothesis testing. 1370 genes were identified as significantly differentially expressed in splenic T-bet^–^ cDC2s and T-bet^+,^ 3624 genes - differentially expressed in splenic cDC1s and T-bet^-^ cDC2s, 2327 genes – in splenic cDC1s and T-bet^–^ cDC2s, 436 genes - between MLN T-bet^–^ cDC2s and T-bet^+^ cDC2s, 1827 genes - between MLN cDC1s and T-bet^+^ cDC2s, 1936 genes - between MLN cDC1s and T-bet^–^ cDC2s. To identify the core gene signature for T-bet^–^ cDC2s and T-bet^+^ cDC2s, we added more stringent conditions to the differential expression analysis between these two subsets in the spleen and in the lymph node, requiring absolute value of log2FoldChange of expression to be above 1.5 and average normalized read counts to be at least 50. This resulted in the list of 316 genes significantly overexpressed in T-bet^+^ cDC2s versus T-bet^–^ cDC2s in the spleen and 142 such genes in the lymph node, with the overlap of 69 genes, and the list of 400 genes significantly overexpressed in T-bet^–^ cDC2s versus T-bet^+^ cDC2s in the spleen and 219 such genes in the MLN, with the overlap of 155 genes. The same processing and differential expression analysis was done for bulk RNA-seq data from YFP^+^ *Rorc(*γ*t)* fate-mapped (Rorγt fm) cDC2s and ILC3s from *Rorc*^*Cre*^*Rosa26*^*lsl-YFP*^ mice, resulting in 3550 differentially expressed genes (Figure S4D). Furthermore, these Rorγt fm cDC2s were normalized and visualized together with T-bet^+^ and T-bet^–^ cDC2s, limited to the genes differentially expressed between T-bet^+^ and T-bet^–^ cDC2s, identified at FDR < 0.01, abs(log2FoldChange) > 1, average normalized read count > 1, resulting in 658 genes (Figure 4H). Library size scaling factors were estimated using DESeq2. Heatmap visualizations of various sets of differentially expressed genes (using z-score per row of log2(library-size normalized count + 1) values) were done using pheatmap v1.0.10. Analysis of association of scRNA-seq data with bulk RNA-seq data was done as follows. Initial raw scRNA-seq read count data over 4420 cells was filtered to contain only genes with total count over all cells > 300, resulting in a matrix of read counts in 6570 genes across 4420 cells. Then counts in each cell were divided by the total read count in that cell, and multiplied by the median across all cells of the total read count per cell. Counts were then transformed using the function log2(count + 1). The resulting transformed normalized counts were used in the following procedure to identify signature genes for each cluster. For each cluster, binomial regression with elastic net regularization and cross-validation was used to predict if a cell belongs to a cluster, given gene expression profiles as features. For this, function cv.glmnet() from package glmnet v2.0-16 (Friedman et al., 2010) was used, with parameters alpha = 0.99, nfolds = 10, type.measure = ”auc.” This analysis resulted in a list of gene coefficients from the model for each cluster. Correlation of these coefficients with library-size-normalized counts from bulk RNA-seq data (for the genes present in both lists) for each sample were used to compare bulk RNA-seq with scRNA-seq data. These were visualized in a heatmap with z-score scaling for each column (Figure 1I).

In order to compare bulk RNA-seq data in mice with previously published human scRNA-seq data from Villani et al. (2017), we took gene signatures of “DC2” and “DC3” cell subpopulations from Villani et al. (2017) as published in a supplementary table of that publication, file aah4573_Supplementary_Tables_1-16.xlsx, panel “Table S3.CD1C subsets.” We extracted these signature genes and found homologous mouse genes using biomaRt v2.28.0. We then plotted CDF of all expressed genes (background) and of these homologs of the “DC2” and “DC3” DC2 signature genes using log2FC of bulk RNA-seq expression between splenic Tbet^+^ cDC2A and Tbet^–^ cDC2B cells, estimated as explained above. The statistical analysis was done using two-sided Kolmogorov-Smirnov test.

#### ATAC-seq analysis

Mouse genome assembly mm10.GRCm38 was used for ATAC-seq read alignments. Gene annotations from GENCODE vM17 were used for all analysis. Analysis was done using R v3.4.0 (2017-04-21) and custom scripts. Reads were aligned to the mouse genome using Bowtie2 v2.2.5 (Langmead and Salzberg, 2012) with parameters “–no-unal -X 1000–no-mixed–no-discordant.” Uniquely aligned reads were extracted using SAMtools v1.9 with parameters “view -h -bS -F 4 -q 20” and sorted and indexed using SAMtools. For peak calling, MACS2 v2.1.1.20160309 (Zhang et al., 2008) was used with parameters “-g hs–nomodel–shift 0–extsize 76–name merged -p 0.1 -B–SPMR–keep-dup 'auto'–call-summits.” Before peak calling, all uniquely aligned reads on strand “+” were shifted by 4bp, and on strand “-” by −5bp. Peak calling was run on all replicates together to obtain a universal list of putative peaks, with peak summits. For each individual replicate, peak calling was run separately with the same parameters. IDR (Li et al., 2011) (https://github.com/nboley/idr, v2.0.3) was then used to determine reproducible peaks in each of the three conditions (cDC1s, T-bet^–^ and T-bet^+^ cDC2s in the spleen). For this, putative peaks from the universal list were used as the oracle set of peaks (parameter “–peak-list”), and lists of peak p values as detected in each individual replicate were used as scores. Peaks at IDR < 0.05 for any pair of replicates of the same condition were called reproducible. Peaks of size 76bp or lower and peaks outside standard chromosomes (chr1-chr19, chrX, chrY) were removed. This resulted in the atlas of 74217 reproducible peaks that contained 177956 peak summits. Each peak was associated with the closest gene according to distance in genomic coordinates, if this distance was not more than 50Kb. Peaks within 2Kb from a transcription start site of any annotated transcript were classified as promoter peaks; the remaining peaks were classified as exonic if overlapping with any exon of any annotated transcript; the remaining peaks were classified as intronic if within a gene body of any annotated gene; the remaining peaks within 50Kb of a gene were classified as intergenic; all the remaining peaks were left unclassified in this classification. Reads in 150bp windows centered around summits (referred to as peaks thereafter and in the main text) were counted using Rsubread v1.22.1. For PCA plot (Figure S4A), variance stabilizing transformation was applied and function plotPCA() from DESeq2 was applied to 500 peaks with the largest variance (parameter “ntop = 2000”). For differential accessibility analysis, DESeq2 v1.18.1 was applied to the read counts, with default FDR adjustment of p values for multiple hypothesis testing. For each pairwise comparison, only the peaks detected as reproducible in the involved conditions were used for the analysis, and furthermore peaks were called significantly more accessible in a condition only if they were also identified as reproducible in that condition. Peaks satisfying these criteria and at FDR < 0.01 and with absolute log2FoldChange > 0.5 were called significantly differentially accessible. In the differential accessibility analysis between T-bet^–^ and T-bet^+^ cDC2s, we identified 2399 peaks significantly more accessible in T-bet^+^ cDC2s and 1130 peaks in T-bet^–^ cDC2s. In the differential accessibility analysis between cDC1s and T-bet^+^ cDC2s, we identified 8711 peaks significantly more accessible in T-bet^+^ cDC2s and 10322 peaks in cDC1s. In the differential accessibility analysis between cDC1s and T-bet^–^ cDC2s, we identified 5055 peaks significantly more accessible in T-bet^–^ cDC2s and 9018 peaks in cDC1s. In the differential accessibility analysis between cDC1s and both T-bet^+^ and T-bet^–^ cDC2s combined, we identified 6392 peaks significantly more accessible in T-bet^+^ and T-bet^–^ cDC2s and 10432 peaks in cDC1s.

Transcription factor binding motifs for *Mus musculus* were downloaded from CIS-BP version 1.02 (Weirauch et al., 2014) via the web interface (compressed archive Mus_musculus_2016_06_01_2-46_pm.zip). For the DNA sequences in 150bp windows around peak summits, script findMotifsGenome.pl from HOMER suite (Heinz et al., 2010) was run with parameters “mm10 -len 8,10,12 -size given -S 100 -N 1000000 -bits -p 10 -cache 1000” in order to identify the significance of presence of each motif in the sequences of the peaks as compared with the background sequences. We limited the analysis to motifs corresponding to expressed transcription factors, defined as those with average library-size-normalized read count across splenic RNA-seq samples > 10 (13601 genes). We focused only on the motifs present in at most 40%, but in at least 1% of the peak summit window sequences. The most significant motif per transcription factor was selected for further analysis (with potentially multiple transcription factors associated with the same motif), if it had HOMER p value < 0.001. This resulted in the list of 124 motifs for further analysis. FIMO version 4.11.2 (Grant et al., 2011) was used to search for motif hits in 150bp windows around the peak summits in the atlas. Hits with *P*-value < 5e-4 were chosen as significant. To determine transcription factors with the strongest association of predicted binding with differential accessibility between a pair of cell states (T-bet^+^ versus T-bet^–^ cDC2s, or cDC1s versus T-bet^+^ and T-bet^–^ cDC2s combined), a supervised linear regression model ***y*** ∼***Xw*** was trained, where ***y*** is a vector of estimated overall log2 fold changes of chromatin accessibility, ***X*** is a feature matrix with rows corresponding to chromatin accessibility peaks and columns corresponding to predicted transcription factors binding sites, and ***w*** is a learned vector of regression coefficients. Only log2 fold changes of chromatin accessibility for 20000 peak summits with the highest normalized counts were used in the model. Scores of transcription factor motifs in windows around peak summits produced by FIMO were used as feature values in ***X***. Lasso regularization with 5-fold cross-validation was used to avoid overfitting. Learned coefficients in this model for each transcription factor motif were used as a proxy of their association with differential accessibility. Fifty motifs with the highest absolute value of the coefficient were visualized as a barplot. Motifs associated with Rara/Rarg and with Rorc were very similar, but still treated as different in the above analysis, and due to lasso regularization, the motif for Rara/Rarg rather than for Rorc was initially picked among the strongest in the analysis of differential accessibility between T-bet^+^ and T-bet^–^ cDC2s. When the analysis was repeated with Rara/Rarg excluded, predictive performance of the model (estimated as Spearman’s rho between real and predicted log2FC values averaged over held-out folds) did not change (rho = 0.28) and Rorc motif essentially substituted that of Rara/Rarg. This version was used for the plot in the main text (Figure 4C). The differential accessibility of the set of peaks associated with the each of the most differentially expressed genes was displayed by “diamond” plots, where the y axis shows the log2 fold change of gene expression between two cell states, diamonds above each gene name correspond to all peaks associated with the gene, and the diamond color corresponds to log2 fold change of accessibility (Figure 4F). For each gene differentially expressed between a certain pair of cell states, overall differential accessibility of its peaks was assessed as follows. A Mann-Whitney U test was used to compare the distribution of all peak accessibility log2 fold changes between these two cell states with those of only the peaks associated with the gene, reporting (qvalue) adjusted *P value*s for the multiple differentially expressed genes tested. This analysis was visualized with a scatterplot where the x axis shows the log2 fold change of gene expression, the y axis shows mean accessibility log2 fold change of the peaks associated with a gene, black indicates differential expression, and red and green indicate significant overall differential accessibility of peaks associated with a gene (Figure 4F).

#### Statistical analysis

Analysis of all data was done with paired two-tailed Student’s t test, one-way or two-way ANOVA with a 95% confidence interval (Prism, GraphPad Software). p < 0.05 was considered significant: ^∗^p < 0.05; ^∗∗^p < 0.01; ^∗∗∗^p < 0.001; ^∗∗∗∗^p < 0.0001. Details as to number of replicates, sample size, significance tests, and value and meaning of *n* for each experiment are included in the Methods or Figure legends. RNA-sequencing experiments were carried out once. Unless otherwise stated, all other experiments were carried out independently at least twice. Mice were non-randomly allocated to experimental groups to ensure equal distribution of genotypes between treatments. Researchers were not blinded as to genotype or treatment during the experiments. No measures were taken to estimate sample size of to determine whether the data met the assumptions of the statistical approaches used. Significance (α) was defined as < 0.05 throughout, after correcting for multiple comparisons. 2 mice from the tamoxifen labeling experiments in Figure 1 were excluded from the analysis because they were found not to have the *R26* allele.

### Data and CODE Availability

The accession numbers for the raw sequencing data reported in this paper are GEO: GSE137710 and GEO: GSE130201. Scripts reproducing the analysis will be available on request.

## Supplemental Information

Table S1. List of EMD/AUROC Genes for Each cDC2 Cluster, Related to Figure 2

Table S2. List of EMD/AUROC Genes for T-bet^+^ cDC2s versus T-bet^–^ cDC2 Clusters, Related to Figure 2

Table S3. List of Core Genes Shared by T-bet^+^ or T-bet^–^ cDC2s across Spleen and MLN, Related to Figure 2

Table S4. List of Differential ATAC-Seq Peaks for Each cDC Subset, Related to Figure 4

Table S5. Gene Ontology (GO) GSEA of AS DC Genes, Related to Figure 7

Table S6. Patient Details for Human Samples, Related to Figures 7 and S7

Table S7. List of EMD Genes for Myeloid Clusters in Human Melanoma, Related to Figures 7 and S7
